# The integrin ligand SVEP1 regulates GPCR‐mediated vasoconstriction via integrins α9β1 and α4β1

**DOI:** 10.1111/bph.15921

**Published:** 2022-08-02

**Authors:** Gavin E. Morris, Matthew J. Denniff, Elisavet Karamanavi, Sarah A. Andrews, Renata B. Kostogrys, Vasiliki Bountziouka, Maryam Ghaderi‐Najafabadi, Noor Shamkhi, George McConnell, Michael A. Kaiser, Laura Carleton, Christine Schofield, Thorsten Kessler, Richard D. Rainbow, Nilesh J. Samani, Thomas R. Webb

**Affiliations:** ^1^ Department of Cardiovascular Sciences University of Leicester and National Institute for Health Research Leicester Biomedical Research Centre, Glenfield Hospital Leicester UK; ^2^ Department of Human Nutrition, Faculty of Food Technology University of Agriculture in Krakow Krakow Poland; ^3^ Horizon Discovery Ltd. Cambridge UK; ^4^ Department of Cardiology, German Heart Centre Munich Technical University of Munich Munich Germany; ^5^ German Centre of Cardiovascular Research (DZHK e. V.), Partner Site Munich Heart Alliance Munich Germany; ^6^ Department of Cardiovascular and Metabolic Medicine & Liverpool Centre for Cardiovascular Science University of Liverpool Liverpool UK

**Keywords:** blood pressure, integrin α4β1, integrin α9β1, SVEP1, vasoconstriction

## Abstract

**Background and Purpose:**

Vascular tone is regulated by the relative contractile state of vascular smooth muscle cells (VSMCs). Several integrins directly modulate VSMC contraction by regulating calcium influx through L‐type voltage‐gated Ca^2+^ channels (VGCCs). Genetic variants in *ITGA9*, which encodes the α9 subunit of integrin α9β1, and *SVEP1*, a ligand for integrin α9β1, associate with elevated blood pressure; however, neither SVEP1 nor integrin α9β1 has reported roles in vasoregulation. We determined whether SVEP1 and integrin α9β1 can regulate VSMC contraction.

**Experimental Approach:**

SVEP1 and integrin binding were confirmed by immunoprecipitation and cell binding assays. Human induced pluripotent stem cell‐derived VSMCs were used in in vitro [Ca^2+^]_i_ studies, and aortas from a *Svep1* ^*+/−*^ knockout mouse model were used in wire myography to measure vessel contraction.

**Key Results:**

We confirmed the ligation of SVEP1 to integrin α9β1 and additionally found SVEP1 to directly bind to integrin α4β1. Inhibition of SVEP1, integrin α4β1 or α9β1 significantly enhanced [Ca^2+^]_i_ levels in isolated VSMCs to Gα_q/11_‐vasoconstrictors. This response was confirmed in whole vessels where a greater contraction to U46619 was seen in vessels from *Svep1* ^*+/−*^ mice compared to littermate controls or when integrin α4β1 or α9β1 was inhibited. Inhibition studies suggested that this effect was mediated via VGCCs, PKC and Rho A/Rho kinase dependent mechanisms.

**Conclusions and Implications:**

Our studies reveal a novel role for SVEP1 and the integrins α4β1 and α9β1 in reducing VSMC contractility. This could provide an explanation for the genetic associations with blood pressure risk at the *SVEP1* and *ITGA9* loci.

## Graphical abstract

Genetic variants in SVEP1 associate with elevated blood pressure. SVEP1 is expressed in VSMCs. SVEP1 plays a novel role in reducing vascular smooth muscle contractility.

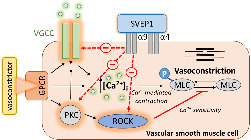

**Abbreviations**
ADAMTS‐7: ADAM metallopeptidase with thrombospondin type 1 motif 7
BSA: bovine serum albumin
BOP: *N*‐(benzenesulfonyl)‐L‐prolyl‐L‐*O*‐(1‐pyrrolidinylcarbonyl)tyrosine
CAD: coronary artery disease
CAEC: coronary artery endothelial cell
CCP: complement control protein
c‐SRC: proto‐oncogene tyrosine‐protein kinase Src
CRISPR: clustered regularly interspaced short palindromic repeats
ECM: extracellular matrix
GFP: green fluorescent protein
GFR: growth factor reduced
IF: immunofluorescence
IHC: immunohistochemistry
IP: immunoprecipitation
iPSC: induced pluripotent stem cell
MBP: mannose binding protein
MLCK: myosin light chain kinase
MLCP: myosin light chain phosphatase
NTC: non‐targeting control
ROCK: Rho A/Rho kinase
siRNA: small interfering RNA
SVEP1: Sushi, von Willebrand factor type A, EGF and pentraxin domain‐containing protein 1
VGCC: L‐type voltage‐gated calcium channel
VSMC: vascular smooth muscle cell
WT: wild‐type

**What is already known?**

- Genetic variants in *SVEP1* associate with elevated blood pressure.

**What does this study add?**

- SVEP1 is a new regulator of vasoconstriction.

**What is the clinical significance?**

- SVEP1 is a potential therapeutic candidate in vascular hypertension.
- Activation of integrin α9β1 could provide a new treatment for vascular hypertension.

## 1 INTRODUCTION

Arterial diseases including hypertension and coronary artery disease (CAD) display a degree of dysregulation in the contractile behaviour of the smooth muscle. Vascular tone is regulated by the relative contractile state of vascular smooth muscle cells (VSMCs) (Brozovich et al., 2016; Webb, 2003). VSMC contraction provides force generation through the phosphorylation of myosin light chain kinase (MLCK), which facilitates interaction between actin and myosin filaments. MLCK is directly phosphorylated by calcium‐bound calmodulin. Increases in intracellular calcium concentrations ([Ca^2+^]_i_) occur via activation of Gα_q_‐GPCRs, leading to PLCβ‐mediated Ca^2+^ ion release from the sarcoplasmic reticulum, and PKC‐mediated activation of L‐type voltage‐gated calcium channels (VGCCs), leading to an influx of extracellular Ca^2+^ ions, with the calmodulin‐dependent MLCK contraction initiated by this elevation in [Ca^2+^]_i_. In addition to activation of MLCK, inhibition of myosin light chain phosphatase (MLCP), via PKC and the RhoA/Rho kinase (ROCK) pathways (Touyz et al., 2018), enables the light chain of myosin to remain phosphorylated and thus prolong contraction. Whilst these central signalling pathways controlling contraction are widely characterised (Brozovich et al., 2016; Touyz et al., 2018; Webb, 2003), modulation of these pathways remains ill‐defined.

Several integrins can directly modulate vascular smooth muscle cell contraction by regulating calcium influx through VGCCs (Mogford et al., 1996; Mogford et al., 1997; Waitkus‐Edwards et al., 2002; Wu et al., 1998; Wu et al., 2001). Within the airway, integrin α9β1 has been specifically identified as preventing exaggerated airway smooth muscle contraction, where conditional knockout of the α9 subunit in airway smooth muscle causes a spontaneous increase in pulmonary resistance in response to several GPCR agonists (Chen et al., 2012). Sushi, von Willebrand factor type A, EGF and pentraxin domain‐containing protein 1 (SVEP1), a high affinity ligand for integrin α9β1 (Sato‐Nishiuchi et al., 2012), is a 390‐kDa secreted extracellular matrix (ECM) protein comprised of sushi (complement control protein [CCP]), von Willebrand factor type A, epidermal growth factor‐like and pentraxin domains (Shur et al., 2006). SVEP1 is a cell adhesion molecule (Gilgès et al., 2000; Sato‐Nishiuchi et al., 2012; Schwanzer‐Pfeiffer et al., 2010; Shur et al., 2006) required for normal development of lymphatic vessels (Karpanen et al., 2017; Morooka et al., 2017) and epidermal differentation (Samuelov et al., 2017). A low‐frequency coding variant rs111245230 (p.D2702G) within SVEP1 associates with elevated blood pressure (BP) (Myocardial Infarction et al., 2016) and CAD (Myocardial Infarction Genetics, 2016).This variant, rs111245230, is situated adjacent to the binding motif through which SVEP1 binds to integrin α9β1. Genetic variants associated with reduced expression of *ITGA9*, which encodes the α9 subunit of integrin α9β1, also associate with increased BP (Evangelou et al., 2018; Levy et al., 2009). Although neither SVEP1 nor integrin α9β1 has a reported role in vasoregulation, direct activation of integrin α4β1, with which integrin α9β1 forms an integrin subfamily (Palmer et al., 1993), can induce VSMC contraction (Waitkus‐Edwards et al., 2002). Recently, two studies investigated the effect of *Svep1* deficiency in relation to the development of atherosclerosis in mice (Jung et al., 2021; Winkler et al., 2020). Notably, these studies, which utilised similar mouse models, detected contrary effects of *Svep1* deficiency with one reporting a reduction in atherosclerosis (Jung et al., 2021) and the other identifying an increase in plaque size (Winkler et al., 2020). The reason for this difference in phenotype is currently unclear. Neither study explored SVEP1 in relation to BP or smooth muscle contraction.

Due to the genetic association between variants in *SVEP1* and *ITGA9* with BP, the described roles for integrins in smooth muscle vasoregulation, including integrin α9β1 in airway smooth muscle, we hypothesised that SVEP1 and integrin α9β1 could regulate vascular smooth muscle contractility. Therefore, in the present study, we analysed the effect of SVEP1 and integrin α9β1 inhibition upon Gα_q_‐GCPR‐mediated VSMC contraction in isolated VSMCs and whole blood vessels.

## 2 METHODS

### 2.1 Cell culture

All cell lines were maintained at 37°C in a 5% CO_2_ incubator. HEK293 wild type cells were maintained in DMEM supplemented with 10% (*v/v*) foetal calf serum (FCS) and integrin α4 over‐expressing cells were maintained in DMEM supplemented with 10% (*v/v*) FCS and 500‐μg·ml^−1^ geneticin.

Human induced pluripotent stem cells (iPSCs) (Cell line GM23720, NIGMS collection from the Coriell Institute for Medical Research, Camden, NJ) were maintained on growth factor reduced (GFR) matrigel‐coated plates in mTeSR™ Plus media (STEMCELL Technologies). Cells were passaged using ReLeSR™ (STEMCELL Technologies) and re‐plated as small clumps of cells at a dilution of 1:10 to 1:20. For SMC differentiation, iPSCs were dissociated with Accutase and plated on GFR Matrigel at a density of 2.5 × 10^4^ cells cm^−2^ in ROCK inhibitor (Y‐27632, 10 μM)‐supplemented mTeSR™ Plus media for 24 h. Media was replaced with STEMdiff™ MIM (STEMCELL Technologies) for 72 h, with media replaced every 24 h. After 72 h, the MIM was replaced with SMC Induction medium consisting of STEMdiff™ APEL‐2 medium (STEMCELL Technologies) supplemented with 50 ng·ml^−1^ VEGF and 25 ng·ml^−1^ BMP4 for 4 days, with media replaced after 2 days. On day 8, cells were dissociated using Accutase and plated on collagen IV (30 μg·ml^−1^ coated wells) in smooth muscle cell growth medium 2 (SMGM2 [Promocell]) supplemented with 10 ng·ml^−1^ PDGF‐BB, 2 ng·ml^−1^ TGFβ, 0.5 ng·ml^−1^ EGF, 2 ng·ml^−1^ bFGF, 5 μg·ml^−1^ insulin and 0.05‐ml·ml^−1^ FCS for a further 14 days. Experiments with cells were carried out between day 32 and day 40.

### 2.2 Generation of SVEP1^−/−^ iPSC lines

An isogenic pair of *SVEP1* GM23720 iPSC line was generated by CRISPR genome editing in collaboration with Horizon Discovery Ltd. A guide RNA targeting GAGACCGCGCCCGGGGCCC *CCGGGAGTATCCCCGCGCCG*CCCGCTCCTGGCGA, a region within exon 1 of Ensembl SVEP1 transcript SVEP1‐003 (ENST00000374469.5) was designed. The underlined highlighted sequence indicates the protospacer adjacent motif and the italic sequence indicates the guide RNA. This guide RNA was co‐transfected into iPSCs with a plasmid expressing CAS9. After transfection, iPSCs were serially diluted into 96 well plates to generate single cell clones. Single cell clones were genotyped by sequencing PCR products generated using primers CAGCCGCTCTGTCTCCAG and AGGAGATGGCAGGGATCTCT.

### 2.3 Cell transfection

iVSMCs were transiently transfected with non‐targeting control (Qiagen siRNA, cat: 1022076), ITGA9 (Qiagen FlexiTube siRNA, cat: S100034272), SVEP1 (ThermoFisher Scientific Stealth siRNA, cat: 1299001) or ITGA4 (Dharmacon SMARTpool of 4 siRNAs, cat: SO‐2757075G) (all 100 nM) using Lipofectamine RNAiMAX (ThermoFisher) diluted in OptiMEM in SMGM2. Media was changed after 24 h, with cells used at 48 h.

### 2.4 Single‐cell Ca^2+^ iVSMC imaging

iVSMCs were loaded with the Ca^2+^‐sensitive dye Fluo‐3, AM (3 μM, 60 min) (ThermoFisher). Cells were maintained at 37°C using a Peltier unit and continually perfused with Krebs–Henseleit buffer (composition in mM: 134 NaCl, 6 KCl, 1 MgCl_2_, 1.2 KH_2_PO_4_, 10 glucose, 10 HEPES, 1.3 CaCl_2_, pH 7.4). Real‐time images were taken using an epifluorescence Nikon Eclipse TE200 microscope (Nikon) (×20 objective) and Volocity 6.1.1 image software (Quorum Technologies). For extracellular Ca^2+^ depletion studies, 10‐mM EGTA was added, and CaCl_2_ was removed from the Krebs–Henseleit buffer, with cells perfused in this buffer for 2 min prior to stimulation. For pharmacological inhibition studies, BOP (*N*‐(benzenesulfonyl)‐L‐prolyl‐L‐*O*‐(1‐pyrrolidinylcarbonyl)tyrosine) (3 μM) or Y‐27632 (10 μM) were added to the cell coverslips 30 min prior to stimulation. Cells were stimulated with vasoconstrictors applied via the perfusion line for 45 s, and Fluo‐3 emission was assessed at ≥520 nm. The maximal fluorescent emission in cells that responded to vasoconstrictor application was measured and then averaged per coverslip to provide a single independent value. [Ca^2+^]_i_ changes are displayed as the fold mean of the fluorescence emission relative to basal fluorescence (*F/F*_*0*_), assigning a value of 0 to *F*_*0*_, to control for sources of variation of baseline fluorescence.

### 2.5 Mouse studies

All animal care and animal experimentation was approved by the local animal ethics committee and performed according to ARRIVE (Animal Research: Reporting of In Vivo Experiments) guidelines (Percie du Sert et al., 2020), and the recommendations made by the *British Journal of Pharmacology* (Lilley et al., 2020), under United Kingdom Home Office Project Licence (P4E9A1CCA). All mice were housed in a specific pathogen‐free facility in an individually ventilated caging system. Mice were group housed wherever possible, and their health status was checked routinely. No mice exhibited any adverse effects. C57BL/6J mice were purchased originally from Charles River, then bred in the Preclinical Research Facility in the University of Leicester, to provide animals for the study. Genetically altered animals, B6N(Cg)‐Svep1tm1b(EUCOMM)Hmgu/J (reporter‐tagged deletion allele, *Svep1* ^+/−^) was purchased from the Jackson Laboratory (Bar Harbor, ME, USA). In accordance with Schedule 1 of the Animals (Scientific Procedures) Act 1986 (U.K.), 13‐ to 24‐week‐old mice of both genders were humanely killed by dislocation of the neck before aortas were removed and used in wire myography experiments.

### 2.6 Wire myography

Aortic ring segments of ~2 mm in length were prepared using a dissecting microscope. Aortic rings where integrin α4 and/or α9 were inhibited were incubated with either BOP or blocking antibodies overnight at 37°C in a 5% CO_2_ incubator in DMEM basal media. Aortic rings were mounted on two intra‐luminal steel wires in a 4‐channel Mulvany–Halpern wire myograph (Danish Myo Technology). Vessels were bathed in a HEPES buffered bath solution containing (mM) NaCl 136, KCl 5, MgSO_4_ 1.2, CaCl_2_ 1.8, glucose 5, mannitol 15, HEPES 10, NaH_2_PO_4_ 0.5 and Na_2_HPO_4_ 0.5 pH 7.4. Isometric tension was continuously recorded using a Powerlab 16/35 AD converter and the LabChart software (LabChart v5, ADInstruments, UK). Vessels were equilibrated, and an optimum static tension of 1.2 mN was observed for a period of at least 50 min before NaCl was reduced to 81 mM and replaced with 60‐mM KCl solution for 10 min, every 10 min for three rounds of high K^+^ solution application to test vascular function. Any vessels that contracted with an amplitude less than 2 mN were excluded from the studies. A single dose of pharmacological inhibitors (nifedipine, 3 μM; BIM (I), 10 μM: BOP, 3 μM; Y‐27632, 10 μM) was added directly to the organ bath, maintained at 37°C, 30 min prior to addition of cumulative concentrations of the vasoconstrictors, U46619 (1–100 nM) or phenylephrine (0.5–200 μM). All vasoconstrictors were added at 10‐min intervals. Aortic rings from the same animals were used for treatment and control experiments. For all experiments, data were expressed as the maximum tension (mN·mm^−1^) generated. Due to genotype requirements, randomisation between groups was not performed when using tissue from *Svep1* ^+/−^ and comparing to wild‐type littermates. Analysis was performed semi‐blinded to treatment and genotype by an independent analyst.

### 2.7 RNA extraction, cDNA synthesis and RT‐qPCR

Total RNA was extracted with RLT buffer and purified using an RNeasy mini kit (Qiagen®) according to the manufacturer's instructions. RNA yield was determined using a NanoDrop ND‐8000 spectrometer. Genomic DNA was removed by DNase I incubation using the RNase‐Free DNase Set (Qiagen®) and RNA was converted to cDNA using SensiFAST cDNA synthesis kit (Geneflow). Quantitative reverse transcription PCR (RT‐qPCR) was performed using SYBR® 3 Green master mix with amplification carried out in triplicate using a Rotor‐Gene® Q (Qiagen®) with each triplicate providing one independent value. Expression levels were calculated using relative standard curve methods and normalised to the reference gene *RPLP0* (Akamine et al., 2007). Primer sequences are listed in Table S1.

### 2.8 Immunohistochemical (IHC) and immunofluorescence (IF) staining

Immunohistochemistry has been conducted to comply with the recommendations made by the *British Journal of Pharmacology* (Alexander et al., 2018). Primary antibodies that were used for IHC and IF are listed in Table S2. Heat‐induced antigen retrieval was performed with Antigen Unmasking Solution, Tris‐Based (Vector, H‐3301) for all antibodies. For IHC staining, endogenous peroxidase activity was blocked in 0.3% H_2_O_2_ in deionised water. Non‐specific binding was reduced by incubation in 2.5% goat serum. Sections were treated with mouse Ig blocking reagent (Vector, MKB‐2213‐1) before application of the primary mouse antibody. Rabbit primary antibody binding was detected with goat anti‐rabbit ImmPRESS HRP goat anti‐rabbit IgG (Vector, MP‐741) and mouse primary antibody binding was detected with Mouse‐on‐Mouse ImmPRESS anti‐mouse Ig reagent (Vector, MP‐2400). Colour was developed with DAB‐substrate chromogen system (Vector, SK‐4100). Images were acquired with a DM2500 Leica microscope (Leica Microsystems).

For IF staining of aortic sections, rabbit primary antibody binding was detected with goat anti‐rabbit IgG (Alexa Fluor‐488), mouse primary antibody binding was detected with goat anti‐mouse IgG (Alexa Fluor‐647) and goat primary antibody was detected with donkey anti‐goat IgG (Alexa Fluor‐594). DAPI was used for nuclei visualisation. Images were acquired using an Olympus FV1000 confocal laser scanning microscope with images analysed using Fiji (Schindelin et al., 2012).

iVSMCs or HUVECs were grown on μ‐Slide 8 well chamber slides (Thistle Scientific) and fixed in 4% PFA. SVEP1, integrin α4 and α9 staining was performed on non‐permeabilised cells. For all other staining, cells were permeabilised in 0.5% Triton‐X. Non‐specific binding was reduced by incubation in 1% bovine serum albumin (BSA), 22.5‐mg ml^−1^ glycine, 0.1% tween‐20 PBS solution, with additional blocking in a 10% goat serum PBS solution. Cells were incubated overnight at 4°C in primary antibody (listed in Table S2) diluted in 10% goat serum. After washing, cells were incubated in 10% goat serum containing complementary secondary antibodies. Nuclei were visualised by DAPI counterstaining. Images were acquired using an Olympus FV1000 confocal laser scanning microscope with images analysed using Fiji (Schindelin et al., 2012).

### 2.9 Flow cytometry

iVSMCs were dissociated using Accutase. CD140^+^ staining was quantified using single cell suspensions incubated using an APC‐direct labelled antibody diluted in flow buffer (BSA (0.5%), EDTA (2 mM), PBS, pH 7.2). Samples were run on a Beckman Coulter Gallios flow cytometer and analysed using Kaluza flow cytometry analysis software (Beckman Coulter).

### 2.10 Western blotting

Western blotting was carried out in compliance with the recommendations made by the *British Journal of Pharmacology* (Alexander et al., 2018). Cells were lysed in modified RIPA buffer (Tris HCl [50 mM], EDTA [1 mM], Halt Protease Inhibitor cocktail [ThermoFisher], pH 7.4). Western Blot Analysis Protein content was measured using the Novex® protein separation kit (ThermoFisher). Equal amounts of protein lysates were separated by SDS‐PAGE before blotting onto nitrocellulose membrane. Membranes were blocked in 5% milk powder, probed with primary antibodies (see Table S2) diluted in 5% milk powder, detected with horseradish peroxidase conjugated secondary antibodies diluted in 5% milk powder and visualised by enhanced chemiluminescence (GE Healthcare). Quantitative signals were derived by densiometric analysis using ImageQuant™ TL on an ImageQuant™ LAS 4000 Luminescent Image Analyzer (Fujifilm). Western blot densitometry values were normalised to the relative quantification of the corresponding intensity of the total protein, and changes in expression were expressed as the fold mean of control cells assigning a value of 1 to the control.

### 2.11 Immunoprecipitation

Constructs expressing ITGA9‐GFP and SVEP1‐FLAG were co‐transfected into HEK293A cells and a construct expressing ITGA4 was transfected into HEK293A or HEK293A cells stably overexpressing SVEP1‐FLAG using Lipofectamine 2000 (ThermoFisher). Forty‐eight hours post transfection the transfected cells were scraped into lysis buffer (mM: 50 Tris‐HCl, 150 NaCl, 1 EDTA, 1% Triton‐X‐100 and 1× phosphatase and protease inhibitors). Lysates were incubated on ice (15 min), sonicated and cleared by centrifugation at 17,000 x *g* for 15 minutes at 4°C. Anti‐FLAG–agarose beads (Sigma Aldrich) were prepared by washing 3× in wash buffer (mM: 50 Tris‐HCl, 150 NaCl and 1 EDTA). Cell lysate was added to the pelleted beads. The IP reactions were incubated for 90 min at 4°C with agitation. The pulled down proteins were denatured from the beads using 25 μl of a solution containing 50% 4× lauryl dodecyl sulphate sample buffer, 45% wash buffer, 5% β‐mercaptoethanol. The ITGA9‐GFP was detected in a western using an anti‐GFP antibody. The ITGA4 protein was detected in a western using an anti‐integrin α4 antibody (primary antibodies listed in Table S2). These westerns were repeated a minimum of 5 times.

### 2.12 Recombinant protein production

Plasmid expressing mannose‐binding protein (MBP)‐tagged CCP21 or CCP22 domains of SVEP1, or MBP alone under the control of an iso‐propyl‐thio‐β‐glactosidase (IPTG) inducible promoter were transformed into *E.coli* BCL21 cells. Transformed cells were grown in lysogeny broth (LB) media containing 100 μg·ml^−1^ ampicillin to an optical density of between 0.6 and 0.8 at 600 nm. Protein expression was induced by addition of 0.5 mM IPTG. Cell culture was pelleted, lysed and sonicated with the lysate cleared by centrifuging. The MBP‐tagged proteins were immunoprecipitated from the cleared cell lysate using amylose beads (New England Biolabs). The protein was eluted from the beads using an affinity purification column with 10‐mM maltose in PBS‐T. The elution buffer was exchanged using spin columns with a molecular weight cut‐off of 30 kDa. Purified protein was run on a 4%–12% Bis Tris gel with protein visualised by Coomassie staining.

### 2.13 Stable cell line generation

To generate integrin‐α4 expressing cells, HEK293A cells were transfected with 2‐μg ITGA4 or SVEP1‐FLAG plasmid using lipofectamine 2000 (ThermoFisher) and selected using 800‐μg·ml^−1^ geneticin 48 h post transfection. Cells were diluted to single cell to isolate individual colonies and clones expressing integrin‐α4 or SVEP1‐FLAG were identified using anti‐integrin α4 or anti‐FLAG antibody respectively (Table S2). To generate an integrin α9‐GFP stable line HEK293A cells were transfected using the NEPA21 Electroporator system (Nepagene). Cells were transfected with 10‐μg integrin α9‐GFP plasmid in OptimMEM. After 48 h, cells were selected using 500‐μg·ml^−1^ geneticin. Cells were diluted to single cell to isolate individual clones, with clones expressing integrin α9‐GFP identified by fluorescent microscopy.

### 2.14 Recombinant protein cell binding assay

The 100‐nM recombinant MBP control, MBP tagged‐CCP21 or MBP tagged‐CCP22 was coated onto a 96 well tissue culture plate. Non‐specific binding was blocked using DMEM containing 10‐mg·ml^−1^ BSA, 10‐mM HEPES. 20,000 HEK293 control (α4/α9^−^), integrin α4β1 (α4β1^+^) or α9β1 overexpressing (α9β1^+^) cells were seeded onto the coated plates in blocking buffer in triplicate with each triplicate providing one independent value. The α4β1^+^ cells were incubated for 3 h and the α9β1^+^ cells were incubated for 30 min at 37°C and incubated at 37°C for 30 min. Plates were washed, fixed with 4% PFA and visualised using DAPI. The number of adhered cells was measured using Fiji (Schindelin et al., 2012). The data were normalised as fold mean over cell adherence to MBP control cells, and changes in adherence are expressed as the fold change over MBP control cells, assigning a value of 1 to the control, to adjust for bound cell numbers.

### 2.15 Data and statistical analysis

The data and statistical analysis comply with the recommendations of the *British Journal of Pharmacology* on experimental design and analysis in pharmacology (Curtis et al., 2018). Each group size was the number of independent values, with the exact group size for each experimental group provided in the figure legends. Group size is the number of independent values, with studies designed to generate groups of equal size, however, outliers were excluded from the single‐cell Ca^2+^ iVSMC imaging studies and the wire myography studies using pre‐defined criteria: In single‐cell imaging, if no cells responded to vasoconstrictor application within a field of view, the value was excluded. In wire myography experiments, contractility was determined by depolarisation in a high K^+^ solution, with vessels that contracted with an amplitude less than 2 mN being excluded from the studies.

**FIGURE 1:**
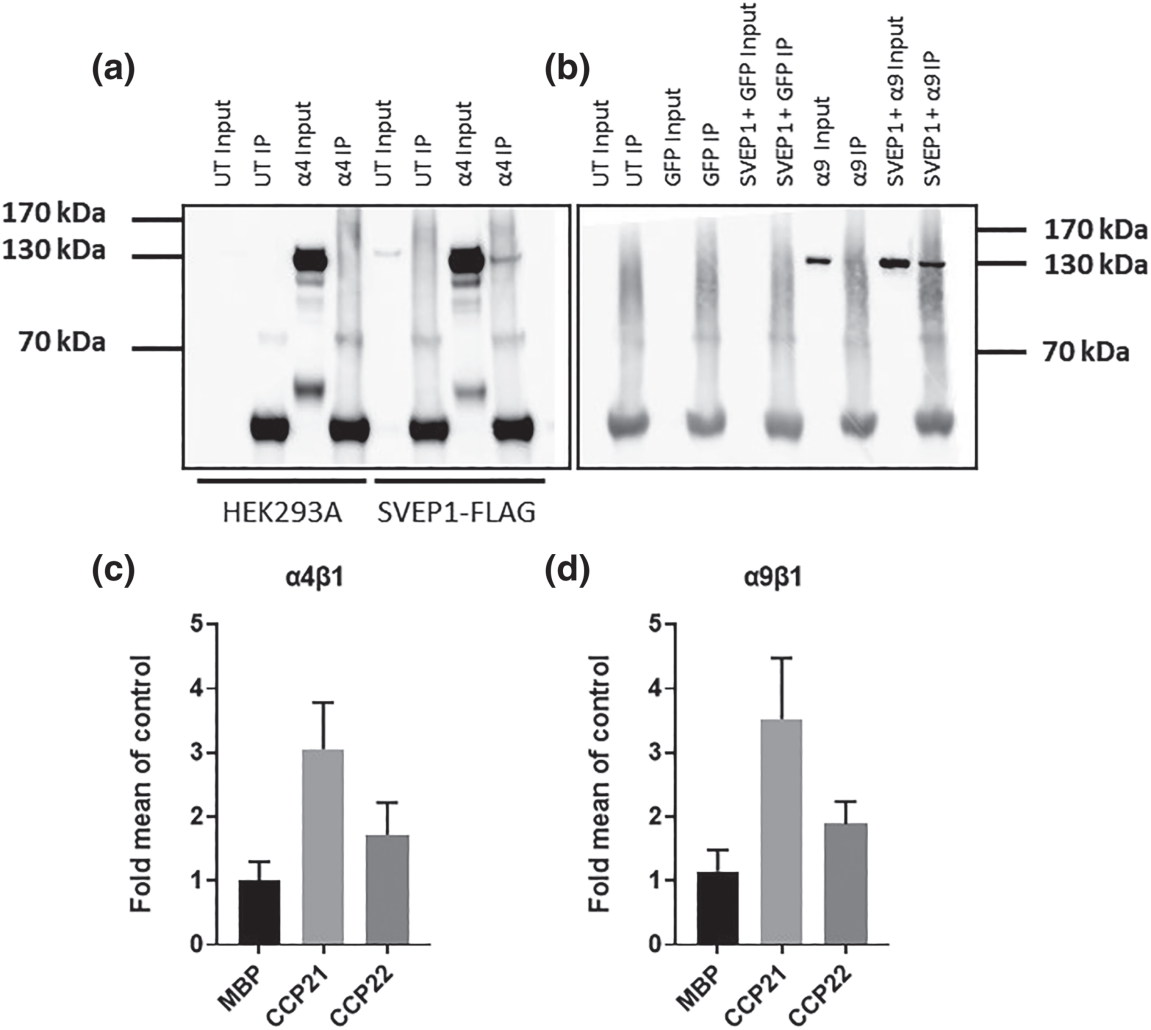
Integrin α4β1 and α9β1 bind to SVEP1 immunoblots with anti‐α4 (a) and anti‐GFP (b) antibodies following immunoprecipitation of protein lysates using anti‐FLAG agarose from HEK293A or HEK293A cells stably overexpressing SVEP1‐FLAG transfected with integrin α4β1 (α4) or HEK293A cells co‐transfected with GFP only control, or GFP‐integrin α9β1 (α9) and SVEP1‐FLAG plasmids. Binding efficiency of HEK293A cells stably overexpressing integrin α4β1 (c) or α9β1 (d) to surface coated with 100 nM mannose binding protein (MBP), or SVEP1 21st or 22nd CCP domain (±SD, *n* = 3). Data are normalised to MBP control to account for variation in cell binding between experiments.

For Figures 1, S1 and S6, the observational and conformational data were not subjected to statistical analysis owing to their small group size (*n* < 5). Statistical analysis performed only for studies where each group size was more than *n* = 5. To reduce unwanted sources of variation derived from different experimental settings, specific data sets were normalised (cell binding assay, Figure 1), single cell imaging (Figures 3, 4, S8 and S9), qPCR (Figures S1 and S6) and western blotting (Figure S6).

Continuous data are presented as mean ± SD. All data transformations are presented as the fold mean over controls. The independent samples Student's *t* test was used to evaluate the differences between two groups. One‐way ANOVA was used to evaluate differences among more than 2 experimental groups. If the overall *F* test was statistically significant, and the variance between groups was constant, we also performed pairwise comparisons using Tukey's multiple comparisons test. To examine the effect of genotype or the application of a targeted antagonist on U46619 (1–100 nM) mediated contraction, we fitted mixed‐effects models. We implemented the restriction maximum likelihood estimation, with random intercepts for the different mice, to account for the within‐mouse variation. Interactions of the genotype with the different levels of concentration were tested. To decide upon the inclusion of the interaction term we used the Bayesian information criterion (BIC). The interaction term was kept in the model if it produced a smaller BIC value compared to a model with no interaction term. For the models with the interaction term, this meant that the genotype effect on vessel tension was not always constant, therefore it varied according to the levels of the U46619 concentration (i.e., dependent on vasoconstrictor concentration: Figures 5c and 6a–d). For the models with no interaction term this meant that the genotype effect on vessel tension was independent of the vasoconstrictor concentration (Figure 5a, 5b, 5d, 6e and S0e). Point estimates are stated in text, while the 95% confidence intervals (95% CI) are plotted in the relevant figures. The models were investigated by inspecting Q‐Q plots and histograms to evaluate the assumption of normality. A value of *P* < 0.05 was considered statistically significant. All statistical analyses were performed using GraphPad Prism 8.0 (GraphPad Software Inc., USA, RRID:SCR_002798) or Stata 16 (StataCorp, 2019).

### 2.16 Materials

BIM (I), carbachol and ET‐1 was supplied by Merck Life Science UK Ltd. (Gillingham, UK) and BOP, phenylephrine and U46619 by Bio‐Techne Ltd. (Abingdon, UK). Nifedipine was supplied by Cayman Chemicals (Ann Arbor, USA) and Y27632 by STEMCELL Technologies (Cambridge, UK).

### 2.17 Nomenclature of targets and ligands

Key protein targets and ligands in this article are hyperlinked to entries in http://www.guidetopharmacology.org and are permanently archived in the Concise Guide to PHARMACOLOGY 2021/22 (Alexander, Christopoulos, et al., 2021; Alexander, Cidlowski, et al., 2021; Alexander, Fabbro, Kelly, Mathie, Peters, Veale, Armstrong, Faccenda, Harding, Pawson, Southan, Davies, Beuve, et al., 2021; Alexander, Fabbro, Kelly, Mathie, Peters, Veale, Armstrong, Faccenda, Harding, Pawson, Southan, Davies, Boison, et al.,  2021).

## 3 RESULTS

### 3.1 SVEP1 binds to integrin α4β1 and α9β1

SVEP1 is a known ligand for integrin α9β1 (Sato‐Nishiuchi et al., 2012), but whether SVEP1 can bind to the closely related integrin α4β1 (Palmer et al., 1993) has not been reported. Using immunoprecipitation, we found SVEP1 to bind to integrin α4 (Figure 1a) and confirmed the ligation of SVEP1 to integrin α9 (Figure 1b). SVEP1 binds to integrin α9β1 through its 21st CCP21 domain (CCP21) (Sato‐Nishiuchi et al., 2012). In addition to demonstrating the direct ligation of SVEP1 to integrin α4, we performed exploratory investigations to determine whether SVEP1 interacts with α4 via the same domain as it interacts with integrin α9 using a cell adhesion assay. We coated tissue culture plastic with 100 nM MBP, MBP‐tagged CCP21 or CCP22 domain peptides. HEK293 cells overexpressing the integrin α4 subunit bound to the CCP21 peptide greater than either MBP or CCP22 control proteins (Figure 1c), with similar results seen for HEK293 cells overexpressing the integrin α9 subunit (Figure 1d).

### 3.2 SVEP1, integrins α4β1and α9β1 are expressed in vascular smooth muscle

**FIGURE 2:**
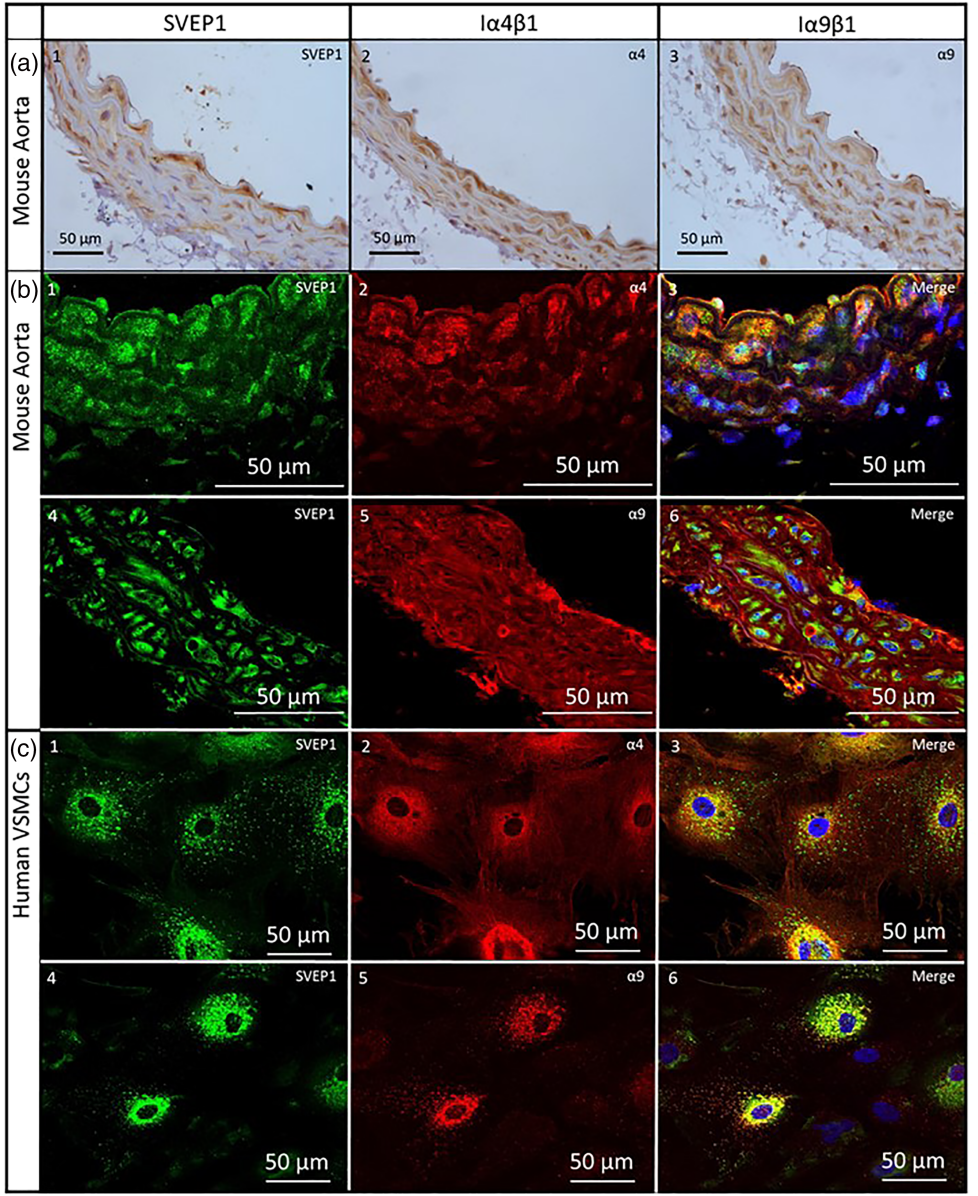
SVEP1, integrin α4β1 and integrin α9β1 expression in vascular smooth muscle. (a) Immunohistochemical staining of SVEP1 (1), integrin α4β1 (2) and integrin α9β1 (3) in mouse aorta sections. (b) Dual fluorescent staining of SVEP1 (1 and 3) and integrin α4β1 (2 and 3), and SVEP1 (4 and 6) and integrin α9β1 (5 and 6) in mouse aorta sections. (c) Dual fluorescent staining of SVEP1 (1 and 3) and integrin α4β1 (2 and 3), and SVEP1 (4 and 6) and integrin α9β1 (3 and 4) in human vascular smooth muscle cells.

**FIGURE 3:**
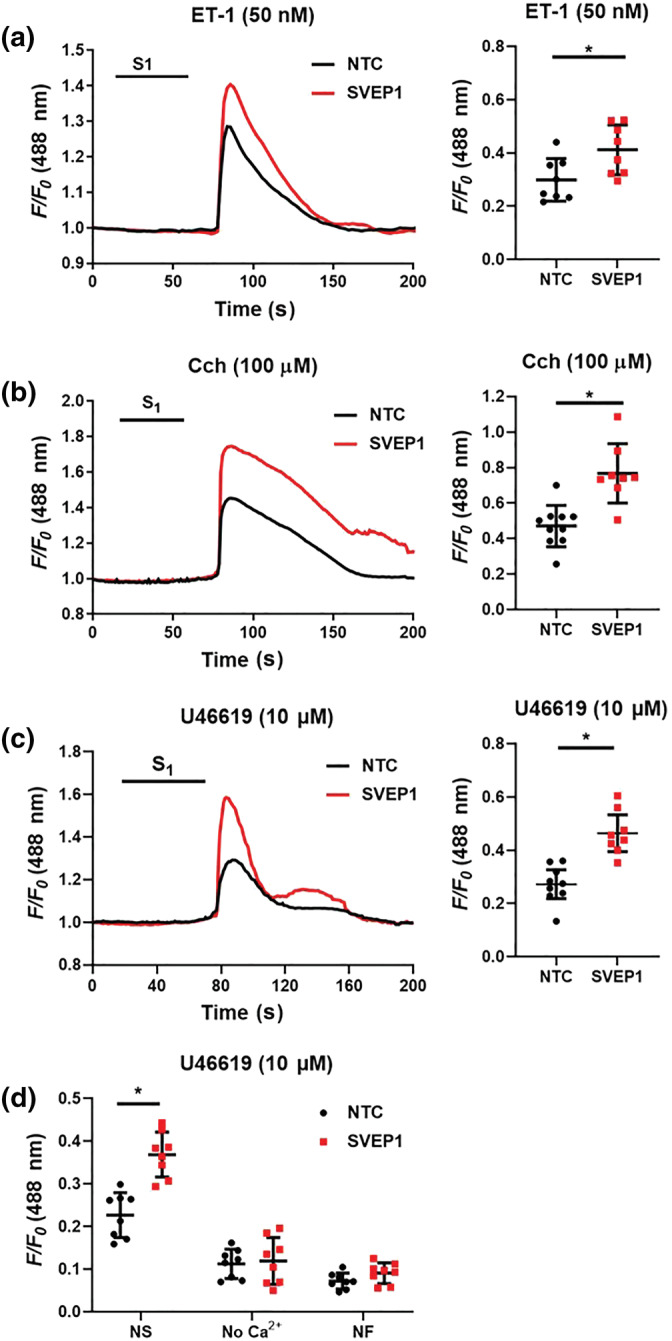
SVEP1 inhibition increases iVSMC [Ca^2+^]_i_ to different vasoconstrictors. iVSMCs were treated with either non‐targeting control (NTC), or SVEP1 siRNA for 48 h prior to Fluo3 loading and vasoconstrictor challenge for 45 s (S1). (a) Mean trace and maximal fluorescence signal (dot plot, F/F0) are shown for ET‐1 (50 nM, *n* = 8), (b) carbachol (Cch; 100 μM), NTC *n* = 10, SVEP1 *n* = 8, and (c) U46619 (10 μM, *n* = 8). (d) Imaging buffer was changed to a zero Ca^2+^ buffer (no Ca^2+^) for 2 min, or incubated in nifedipine (NF, 3 μM) for 30 min prior to U46619 challenge (10 μM, *n* = 8); NS, non‐stimulated control. Data presented are individual values with means ± SD. *P < 0.05, significantly different as indicated; unpaired *t* test.

We explored the gene expression of *SVEP1*, *ITGA4* and *ITGA9* in both endothelial cells and VSMCs, the primary resident cell types of the blood vessel wall. Each gene was expressed in both cell types with *SVEP1* (Figure S1a) and *ITGA4* (Figure S1b) more highly expressed in VSMCs and *ITGA9* expression higher in endothelial cells (Figure S1c), in keeping with the previous atherosclerosis studies (Jung et al., 2021; Winkler et al., 2020). Subsequent protein analysis revealed expression of SVEP1, integrin α4β1 and integrin α9β1 within the arterial wall, with all three proteins localised to VSMCs within the media layer of the arterial wall (Figures 2a, 1–3, and S2). Immunofluorescent dual staining showed SVEP1 to be in close proximity with integrin α4β1 (Figure 2b, 1–3) and integrin α9β1 (Figure 2b, 4–6) in mouse aorta and isolated human VSMCs (Figure 2c, 1–3 and 4–6, respectively). SVEP1 protein was found to be in close proximity to both integrin α4β1 and integrin α9β1 at low levels in isolated HUVEC cells (Figure S3). Relevant staining controls are shown in Figure S4.

### 3.3 Development of a human VSMC in vitro platforms for SVEP1 vasoconstrictive investigations

A limiting factor in smooth muscle contraction experiments is the loss of membrane channels and GPCRs within days of culturing following tissue extraction (Halayko et al., 1996; Ihara et al., 2002; Widdop et al., 1993). To overcome this issue, we developed a human iPSC‐derived vascular smooth muscle cells (iVSMC) model with iPSCs differentiated into a mesodermal phenotype as a monolayer, prior to differentiation into specialised VSMC phenotype (Maguire et al., 2017). iPSC pluripotency gene expression is stopped by day 4 (Figure S5b, 1 and 5). Cells differentiate into primitive streak cells (days 2–4, Figure S5b, 2 and 6) and mesodermal progenitors (days 3–6, Figure S5b, 3), with 94% of cells CD140^+^ at day 8 (Figure S5b, 7). After a further 12 days culture in TGFβ and PDGF supplemented media, the iVSMCs express a panel of smooth muscle contractile markers (Figure S5c), reliably physically contract a collagen gel (Figure S5d, 1) and display an increase in [Ca^2+^]_i_ in response to a panel of GPCR vasoconstrictors (Figure S5d, 2), compared to the limited contractile responses seen in cultured primary human VSMCs (Figure S5d, 3).

To interrogate the role of SVEP1, integrin α4β1 and α9β1 in VSMC contraction we used two complementary methods. Gene expression of *SVEP1*, *ITGA9* and *ITGA4* were knocked down using siRNA in differentiated iVSMCs. We achieved a knockdown efficiency between 60% and 90% at the RNA level, with protein knockdown confirmed for integrin α4 and α9 by western blotting and immunofluorescence, and SVEP1 by immunofluorescence alone (Figure S6). We were unable to detect a band of the correct molecular weight to reliably quantify SVEP1 protein expression. In addition to siRNA depletion of *SVEP1*, we generated *SVEP1* ^*−/−*^ knockout iPSCs using CRISPR‐Cas9, which contain a 1 base pair deletion at position 130 in the coding sequence within exon 1 of *SVEP1* (Figure S7). This isogenic pair of iPSCs were then differentiated into iVSMCs and used in [Ca^2+^]_i_ experiments.

### 3.4 SVEP1 and integrin α4 or α9 deficiency enhances VSMC [Ca^2+^]_i_ elevation

**FIGURE 4:**
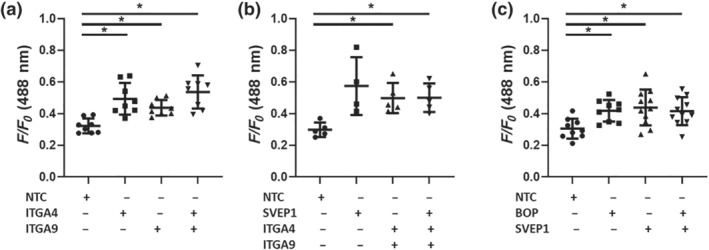
Simultaneous inhibition of SVEP1 and integrin α4 or α9 does not induce additional [Ca^2+^]_i_ elevation in iVSMCs were treated with non‐targeting control (NTC), ITGA4, ITGA9 or SVEP1 siRNA for 48 h, or the dual integrin α4β1‐α9β1 inhibitor BOP for 2 h prior to Fluo3 loading and ET‐1 (50 nM) challenge for 45 s. Maximal fluorescence signal (F/F0) are shown (a) *n* = 8, (b) NTC, ITGA4, ITGA9 *n* = 5, SVEP1 *n* = 4, (c) NTC, BOP *n* = 9, SVEP1 *n* = 10, SVEP1 and BOP *n* = 11. Data presented are individual values with means ± SD. *P < 0.05, significantly different as indicated; one‐way ANOVA followed by Tukey's post hoc test.

*SVEP1* siRNA treated isolated iVSMCs showed significant increases in cytosolic [Ca^2+^]_i_ to several vasoconstrictors that signal via different GPCRs including endothelin (ET)‐1 (Figure 3a), carbachol (Figure 3b) and U46619 (Figure 3c) compared to non‐targeted control (NTC) siRNA transfected cells. This effect was confirmed in *SVEP1* ^*−/−*^ iVSMCs where maximal [Ca^2+^]_i_ elevation to ET‐1 (Figure S8a) and carbachol (Figure S8b) were also significantly enhanced compared to isotype control iVSMCs. Increases in intracellular Ca^2+^ occur through Ca^2+^ release from the sarcoplasmic reticulum and via an influx of extracellular Ca^2+^ through VGCCs (Brozovich et al., 2016; Nelson & Quayle, 1995; Touyz et al., 2018; Webb, 2003). To investigate the source of the increased [Ca^2+^]_i_, extracellular Ca^2+^ was depleted in the imaging buffer, or the VGCC antagonist nifedipine was added prior U46619 application. Both removal of extracellular Ca^2+^ and VGCC blockage minimised [Ca^2+^]_i_ accumulation upon U46619 stimulation in both *NTC* and *SVEP1* siRNA treated cells (Figure 3d), indicating the elevation of [Ca^2+^]_i_ was primarily achieved through the influx of extracellular Ca^2+^. Inhibition of either integrin α4β1 or α9β1 using siRNA caused enhanced iVSMC [Ca^2+^]_i_ elevation to ET‐1 (Figure 4a), whilst simultaneous inhibition of integrin α4β1 and α9β1 did not cause any additional [Ca^2+^]_i_ increase (Figure 4a). Similarly, *SVEP1* deficiency and blocking either integrin α4β1 (Figure S9a), integrin α9β1 (Figure S9b) or integrin α4β1 and α9β1 dual inhibition using siRNA (Figure 4b) or the dual integrin α4β1/α9β1 inhibitor BOP (Pepinsky et al., 2002) (Figure 4c) did not cause additional ET‐1‐mediated [Ca^2+^]_i_ elevation compared to cells treated with *SVEP1* siRNA alone. Similar results were seen in iVSMCs stimulated with carbachol (Figure S9c) and was confirmed in ET‐1‐stimulated *SVEP1* ^*−/−*^ iVSMCs treated with BOP (Figure S9d). These data show that SVEP1 reduces iVSMC Ca^2+^ release to several Gα_q/11_ agonists via integrin α4β1 and α9β1.

### 3.5 SVEP1‐integrin α4/9 signalling inhibits whole vessel contraction

Perinatal mortality is observed in *Svep1* null mice, with mice displaying oedema at E18.5 (Morooka et al., 2017). *Svep1* ^+/−^ mice have reduced *Svep1* mRNA expression in the lung and aorta (Winkler et al., 2020) and have no gross phenotypic effects (Morooka et al., 2017; Winkler et al., 2020) and were used for ex vivo analysis of vessel contraction.

**FIGURE 5:**
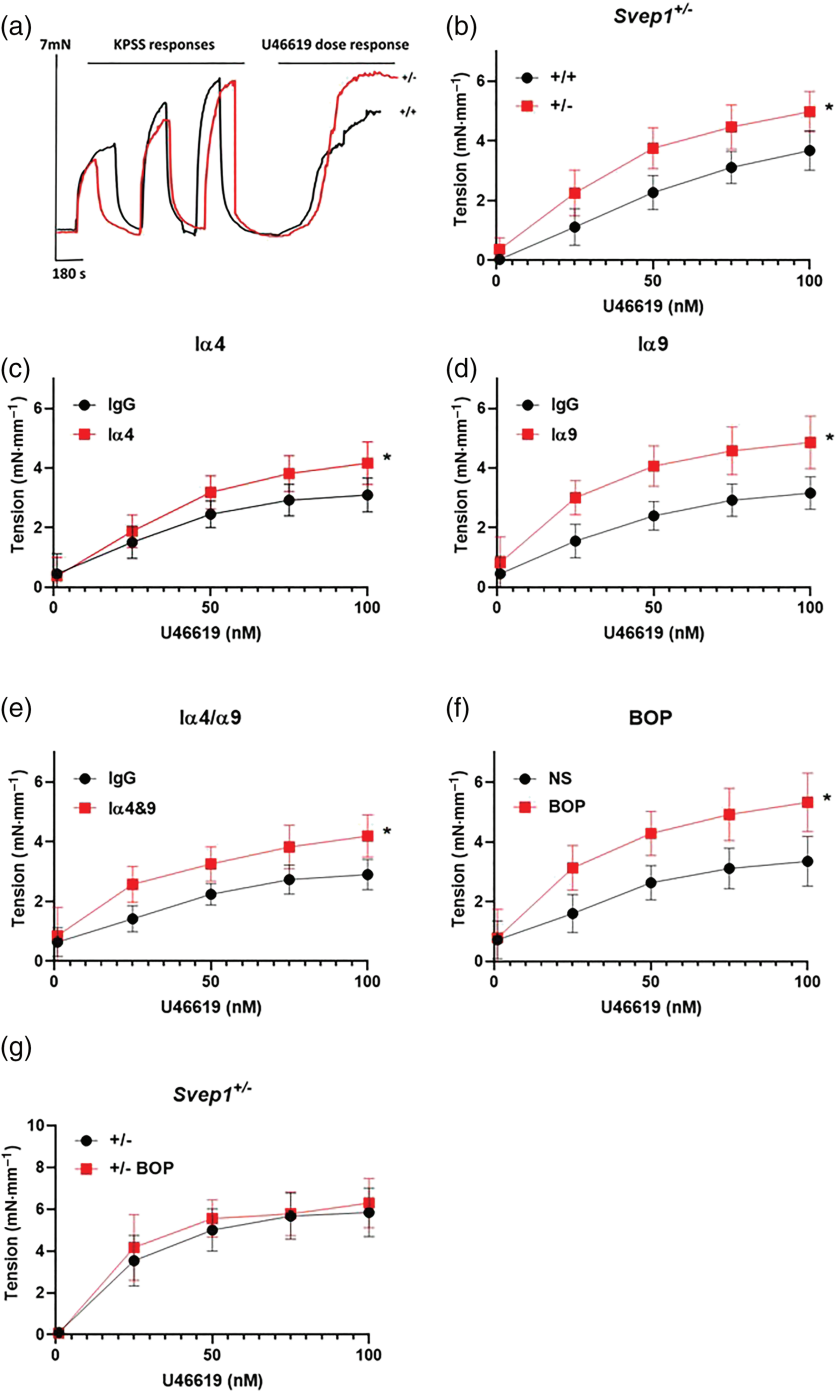
SVEP1 or integrin α4/9 inhibition enhances blood vessel contraction. (a) Typical traces showing force generation of aortas from *Svep1* ^+/−^ (+/−) and littermate control (+/+) mice stimulated with KPSS (high K^+^ physiological salt solution, 60 mM KCl) prior to application of U46619 (1‐100 nM). (b) Aortas from *Svep1* ^+/−^ mice were stimulated with U46619 (+/+ *n* = 11, +/− *n* = 13) and force generation was recorded by wire myography. (c) Aortas from C57BL/6J mice were incubated overnight with an integrin α4 (10 μg·ml^−1^) (IgG *n* = 10, ITGA4 *n* = 10), (d) integrin α9 (10 μg·ml^−1^) (IgG *n* = 10, ITGA9 *n* = 10), (e) a combination of both integrin α4 & α9 blocking antibodies (IgG *n* = 11, 4 and 9 *n* = 12) or (f) the dual integrin α4 and α9 inhibitor BOP (3 μM) (NS *n* = 6, BOP = 10) prior to U46619 application. (g) Aortas from *Svep1* ^+/−^ mice were incubated overnight with BOP (+/− *n* = 10, +/− BOP *n* = 10) and force generation was recorded. Data presented are means with 95% confidence intervals. *P < 0.05, significantly different as indicated; mixed‐effect models.

Vessels from *Svep1* ^*+/−*^ mice showed a significantly higher contraction to U46619 (Figure 5a,b) and phenylephrine (Figure S10a), compared to littermate controls. Incubation of vessels from C57BL/6J mice with an integrin α4 blocking antibody (10 μg·ml^−1^, MCA1230Ga, Figure 5c), or an integrin α9 blocking antibody (10 μg·ml^−1^, 55A2C, Figure 5d) significantly enhanced contraction to U46619. Simultaneous blocking of integrin α4β1 and α9β1 using blocking antibodies (Figure 5e) or BOP (3 μM, Figure 5f) caused a significant increase in vessel tension but did not enhance contraction compared to inhibition of individual integrins in isolation. Inhibition of integrin α4/α9 using BOP did not enhance contraction in *Svep1* ^*+/−*^ mice (Figure 5g).

### 3.6 VGCCs and PKC regulate SVEP1‐integrin α4/α9 inhibition of smooth muscle contraction

**FIGURE 6:**
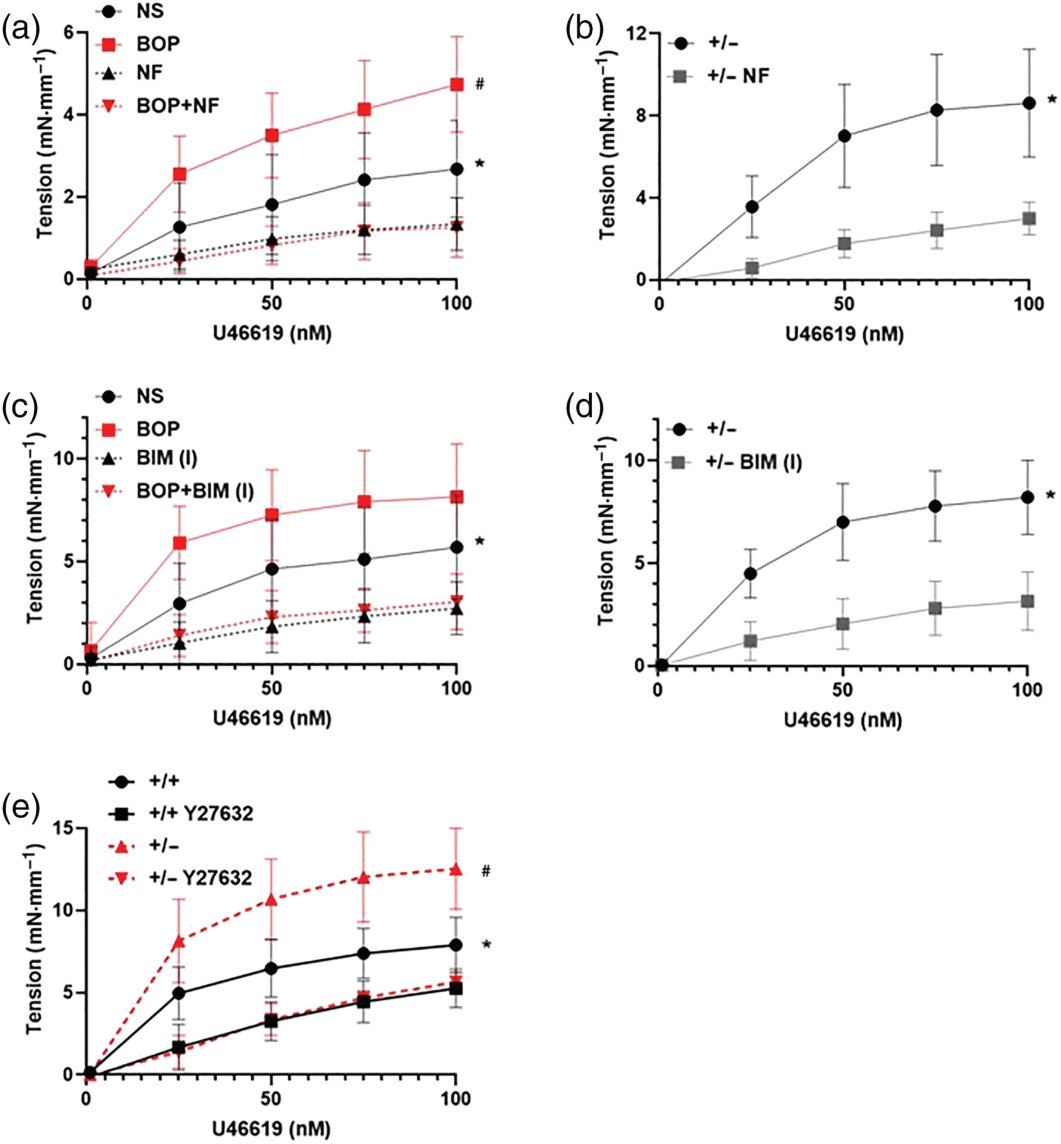
Integrin α4/9 regulates blood vessel contraction via Ca^2+^ influx through VGCCs in a PKC and ROCK dependent manner (a) aortas from C57BL/6J mice were incubated with the dual integrin α4/9 inhibitor BOP overnight and incubated with the VGCC blocker nifedipine (NF) for 30 min prior to U46619 application and force generation was recorded; NS, non‐stimulated control. Data presented are means, with 95% confidence intervals; *n* = 7. * *P* < 0.05, NS significantly different from NF; # *P* < 0.05, BOP significantly different from BOP + NF; mixed effect models. (b) Aortas from *Svep1* ^+/−^ mice were incubated with NF. Data presented are means, with 95% confidence intervals; *n* = 6. *P < 0.05, significant effect of NF; mixed effect models. (c) Aortas from C57BL/6J mice were incubated with BOP overnight and incubated with the PKC inhibitor BIM (I) for 30 min prior to U46619 application; NS, non‐stimulated control. Data presented are means, with 95% confidence intervals; *n* = 7. * *P* < 0.05, NS significantly different from BIM (I), # *P* < 0.05, BOP significantly different from BOP+BIM (I); mixed effect models. (d) Aortas from *Svep1* ^+/−^ mice were incubated with BIM (I). Data presented are means, with 95% confidence intervals;.*n* = 9. *P < 0.05, significant effect of BIM (I); mixed effect models. (e) Aortas from *Svep1* ^+/−^ mice (+/−) or littermate control mice (+/+) were incubated with the ROCK inhibitor Y27632 for 30 min prior to U46619 application and force generation was recorded. Data presented are means, with 95% confidence intervals; *n* = 7. *P < 0.05, significant effect of Y2673 in control mice, # *P* < 0.05, significant effect of Y2673 in *Svep1* ^+/−^ mice; mixed effect models.

Aortas from C57BL/6J were either pre‐incubated with BOP (Figure 6a) or integrin α4 and α9 blocking antibodies (Figure S10b) in the presence or absence of the VGCC inhibitor nifedipine (3 μM) prior to U46619 stimulation. VGCC inhibition significantly lowered both normal U46619‐mediated vessel contraction (Figure 6a), and the enhanced contraction caused by integrin α4/9 inhibition using BOP (Figure 6a). In *Svep1* ^*+/−*^ mice, inhibition of VGCCs also significantly reduced U46619‐mediated contraction (Figure 6b). VGCCs activity is regulated by protein kinase C (PKC) (Ringvold & Khalil, 2017). Inhibition of PKC using bisindolylmaleimide I (BIM (I), 10 μM) significantly reduced normal U46619‐mediated contraction (Figure 6c), and the enhanced contraction caused by integrin α4/9 inhibition (Figure 6c). BIM (I) inhibition of PKC also significantly reduced U46619‐mediated contraction in *Svep1* ^*+/−*^ mice (Figure 6d). These results show that SVEP1 regulation of GPCR‐mediated contraction occurs through regulating PKC‐mediated VGCC Ca^2+^ influx into the vessel.

To ensure the modulation in contractile responses elicited by SVEP1 and integrins α9β1/α4β1 is via receptor‐mediated Ca^2+^ influx through VGCCs, and not a receptor‐independent direct activation of VGCCs, we compared vessels stimulated with extracellular KCl between aortas from *Svep1* ^*+/−*^ mice and littermate controls (Figure S10c) and aortas from C57BL/6J pre‐incubated with BOP (Figure S10d) stimulated with extracellular KCl. No alterations in contractile responses were detected between both groups, confirming that SVEP1 did not directly affect VGCC activation and the observed alterations in U46619‐mediated contraction is via receptor‐mediated signalling.

### 3.7 ROCK regulates SVEP1 inhibition of smooth muscle contraction

**FIGURE 7:**
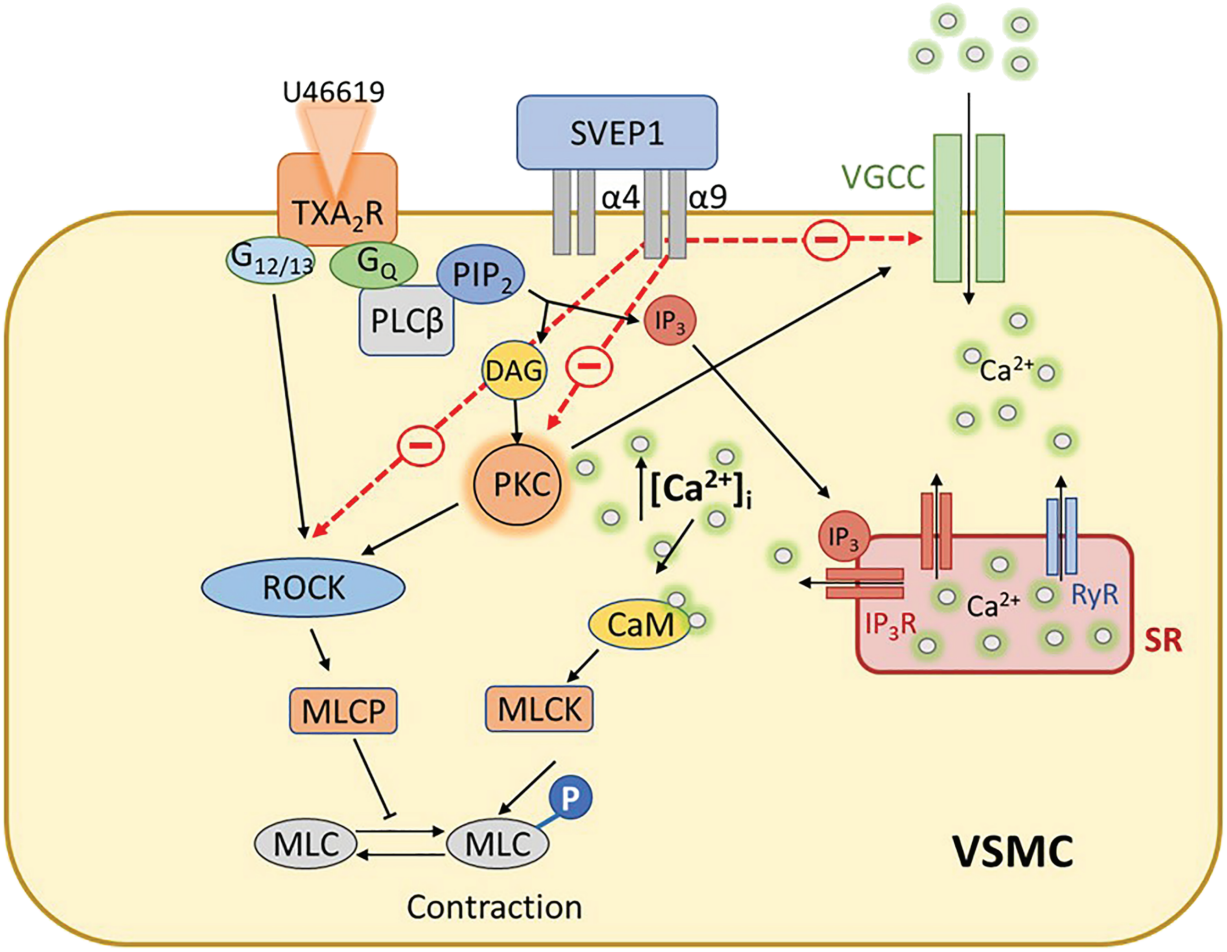
Diagram of proposed model of how SVEP1 regulated GPCR‐mediated vasoconstriction. U46619 binds to TXA_2_ receptors (TXA2R) to activate Gα_q_ and G_12/13_ signalling. Gα_q_ activates PLCβ, which hydrolyses PIP_2_ into DAG and IP_3_. Binding of IP_3_ to the IP_3_ receptors (IP_3_R) on the SR induces Ca^2+^ release from stores. DAG activated PKC promotes the opening of VGCCs to initiate Ca^2+^ influx into the cell. Ca^2+^‐bound CaM activates MLCK, which phosphorylates MLC leading to contraction. Activation of PKC and G_12/13_ also activates ROCK, which inhibits MLCP, promoting further activation of MLC and contraction. SVEP1 regulates contractility of VSMC via integrins α4/α9 by interacting with both calcium‐dependent pathways that reduce PKC activity and the influx of extracellular Ca^2+^ through VGCCs, and calcium sensitisation via ROCK. Abbreviations: α4, integrin α4β1; α9, integrin α9β1; CaM, calmodulin; DAG, diacylglycerol; IP_3_, inositol triphosphate; MLC(K)(P), myosin light chain (kinase)(phosphatase); PIP_2_, phosphatidylinositol diphosphate; PKC, protein kinase C; PLCβ, phospholipase C β; ROCK, Rho A/Rho kinase; SR, sarcoplasmic reticulum; VGCCs, voltage gated calcium channels; VSMC, vascular smooth muscle cell

In addition to regulating VGCC‐PKC mediated Ca^2+^‐dependent vasoconstriction we investigated whether calcium sensitization mediated the regulation of VSMC contraction by SVEP1. ROCK signalling can inhibit MLCP activity to prolong MLC activity, maintaining VSMC contraction (Loirand & Pacaud, 2010). Pharmacological inhibition of ROCK (Y27632, 10 μM) significantly lowered both U46619‐mediated control vessel contraction (Figure 6e) and the enhanced contraction seen in *Svep1* ^*+/−*^ mouse aortas (Figure 6e). SVEP1 reduces VSMC contraction by acting upon Ca^2+^‐dependent signalling and PKC to alter Ca^2+^ influx through VGCCs, and reduced calcium sensitivity via ROCK (Figure 7).

## 4 DISCUSSION

The data presented here represents the first investigation of SVEP1 and integrin α9β1 in vasoconstriction. SVEP1 was found to bind to integrin α9β1 and for the first time, the closely related integrin α4β1. Cell adhesion studies suggest that SVEP1 binds to integrin α4β1 through its CCP21 domain. Within the vasculature and in isolated VSMCs, we found expression of SVEP1, integrin α4β1, and α9β1 to be predominantly localised within the media layer, confirming previous data (Jung et al., 2021). Due to the genetic association between *SVEP1* and *ITAG9* with BP, and the reported regulatory role for integrin α9β1 in airway contraction, we investigated whether SVEP1 could play a regulatory role in VSMC contraction via integrins α4β1/α9β1.

Our single cell [Ca^2+^]_i_ analysis showed that inhibition of SVEP1 or integrins α4/α9 increased [Ca^2+^]_i_ in response to several vasoconstrictors in iVSMC, suggesting a general regulatory effect upon receptor‐mediated [Ca^2+^]_i_ elevation. Subsequent whole vessel studies where integrins α4/α9 were inhibited or SVEP1 levels were reduced in *Svep1* ^*+/−*^ mice, contractile force was also enhanced to either phenylephrine or U46619 application. SVEP1 or integrin α4/α9 inhibition had no effect on direct vessel contraction to smooth muscle depolarisation by KCl, indicating the regulatory role of SVEP1 is specific to receptor‐mediated vasocontraction. We found similar increases in Ca^2+^ levels upon inhibition of integrin α4β1 or α9β1 and no additional alterations in [Ca^2+^]_i_ were detected with co‐inhibition of SVEP1 and the integrins in iVSMCs. Comparable results were observed in whole vessel contraction. These data suggest that the effect of SVEP1 on contraction is solely via integrin signalling and also indicates a level of redundancy between integrin α4β1 or α9β1 or a ceiling effect of SVEP1 inhibition upon vessel contraction. In the airway, ligation of integrin α9β1 can prevent GPCR‐mediated airway hyperresponsiveness (Chen et al., 2012), a phenotype comparable to the vascular role for integrin α9β1 and SVEP1 identified here. The physiology of airway smooth muscle cells differs from that of VSMCs, and integrin α9β1 instead regulates Ca^2+^ release from intracellular stores (Chen et al., 2012), meaning the downstream signalling events are likely to be different.

To determine the underlying SVEP1‐integrin mediated regulation of GPCR‐signalling, we focused upon U46619 vasoconstriction mediated via the thromboxane A_2_ (TXA_2_) receptor (TP receptor) that, in addition to coupling with Gα_q11_, also couples with G_12/13_, which activates ROCK, causing phosphorylation of MLCP and increased Ca^2+^ sensitivity in VSMCs (Pang et al., 2005). Previous studies have uncovered various signalling pathways that control TP receptor‐mediated arteriole contraction, suggesting the relative importance of the pathways could be tissue and species specific. In bovine pulmonary arteries, contraction was mainly ROCK‐mediated with little evidence of VGCC involvement (Alapati et al., 2007), whereas rat pulmonary artery contraction was PKC‐VGCC mediated with little evidence of ROCK involvement (Cogolludo et al., 2003). Contraction in rat caudal arteries involved VGCCs and ROCK, with little evidence of the requirement for PKC (Wilson et al., 2005). In mouse renal (Yan et al., 2019), mouse coronary (Jiang et al., 2021) and porcine coronary arteries (Nobe & Paul, 2001) each required the Ca^2+^ sensitive VGCCs, ROCK, and PKC all to be involved in the vasoconstriction mediated by U46619.

In mouse aorta, we investigated both Ca^2+^‐dependent and Ca^2+^ sensitisation contractile mechanisms by inhibiting VGCCs and ROCK respectively, and additionally PKC, a central mediator of both mechanisms. Our data suggest that, in the aorta, U46619 initiates vessel contraction through both VGCC‐mediated Ca^2+^‐dependent contraction and via ROCK kinase‐mediated Ca^2+^‐sensitisation dependent contraction (Figure 7) as described in other arterioles (Jiang et al., 2021; Nobe & Paul, 2001; Yan et al., 2019). Furthermore, the similar inhibition of U46619‐induced contraction in *Svep1* ^*+/−*^ mice or integrin α4β1/α9β1 inhibited mice to controls, suggested that the vasoregulatory effect was mediated via the same pathway, indicating *Svep1* deficiency is mediated via PKC, VGCC and ROCK (Figure 7). In these experiments, we used HEPES buffered solution to bathe the vessels. It is conceivable that the environmental conditions used in myography could affect vessel responses to vasoconstrictor application. However, we found our aortic contractile responses to be comparable with other studies stimulating aortic segments to U46619 when bathed in Krebs' buffer gassed continuously with 95% O_2_ and 5% CO_2_ (Heinze et al., 2014; Jiménez‐Altayó et al., 2020).

Several studies have administered synthetic ligands to mimic important vasoactive ECM fragments, which are otherwise un‐exposed within the full‐length ECM molecules (Davis, 2010). Dysregulation of the ECM is linked to several vascular‐associated diseases including CAD (Galis & Khatri, 2002), heart failure (Westman et al., 2016), and stroke (Hill & Nemoto, 2015). SVEP1 is a substrate of the protease ADAMTS‐7 (Kessler et al., 2015), which also include genetic variants associated with CAD (Coronary Artery Disease Genetics, 2011; Nelson et al., 2017) and BP (Warren et al., 2017), and contains the linear peptide sequences Arg‐Gly‐Asp (RGD) and Leu‐Asp‐Val (LDV) sequences. Upon direct ligation to integrin αvβ3 (Mogford et al., 1996; Wu et al., 1998), RGD inhibits VGCC current in smooth muscle, whilst the binding of LDV to integrin α4β1 (Waitkus‐Edwards et al., 2002), α5β1 (Mogford et al., 1997; Wu et al., 1998; Wu et al., 2001) and integrin α7β1 (Kwon et al., 2000) causes Ca^2+^ mediated smooth muscle contraction. It would be interesting to determine whether SVEP1 breakdown products also have altered vasoregulatory effects.

Recent studies produced conflicting data concerning *Svep1* deficiency in relation to the development of atherosclerotic plaques in mice (Jung et al., 2021; Winkler et al., 2020). Both investigations identified *SVEP1* expression in VSMCs and endothelial cells within blood vessels but found opposing effects of SVEP1 in inflammatory cell recruitment, possibly highlighting distinct functions for SVEP1 in different cell‐types. However, the cause for the phenotypic difference in atherosclerosis is unclear. In our data, *Svep1* deficiency increases contraction and would support SVEP1 as a protective molecule for reducing BP, which might contribute to atheroprotection. Notably, human genetic studies have identified associations between variants in both SVEP1 (Myocardial Infarction et al., 2016) and integrin α9β1 and BP (Evangelou et al., 2018; Levy et al., 2009). SVEP1 and integrins α4β1 and α9β1, as new mediators of GPCR‐mediated vasoconstriction, provide a novel pathway whose activation could provide new therapeutic targets in vascular hypertension. Further studies should investigate whether the disease‐associated variants alter the contractile response of VSMCs and resistance vessels to contribute to an altered BP.

In conclusion, we have described for the first time how the ECM protein SVEP1 lowers VSMC contractility, via integrin α4β1 and/or α9β1, by influencing pathways that reduce Ca^2+^ influx through VGCCs and reduced calcium sensitivity, providing a new link between the extracellular environment and VSMC contraction.

## CONFLICT OF INTEREST

The authors declare no conflicting interests.

## AUTHOR CONTRIBUTIONS

G.E.M and T.R.W conceived the study and participated in the overall design, and coordination of the study. G.E.M designed and performed in vitro experiments. S.A.A performed the immunoprecipitation and cell binding studies. E. K and R.B.K performed IHC analysis. E. K and M.J.D performed wire myography experiments, with R.D.R and T. K providing support and supervision for the ex vivo models, and V. B providing statistical support. G.E.M, G. M, N.M.G, N. S, M.A.K, L. C, C. S and T.R.W designed and generated the SVEP1 knockout iPSC lines. G.E.M., N.J.S. and T.R.W supervised the overall project. G.E.M and T.R.W wrote the manuscript. All authors reviewed the manuscript.

## Supporting information


**Table S1:** Primer sequences
**Table S2:** Antibody suppliers and catalogue numbers

**Figure S1:** **SVEP1 and integrin expression in the vasculature**
(A) SVEP1, (B) ITGA4 and (C) ITGA9 mRNA expression was measured using qRT‐PCR in human coronary artery endothelial cells (CAEC) and vascular smooth muscle cells (VSMC). Results are normalised to the reference gene RPLPO. Data are represented as means± SD,n = 3.
Figure S2.SVEP1 integrin α4β1 and integrin α9β1 are localised to smooth muscle cell layer
Dual fluorescent staining of SVEP1 and smooth muscle α‐actin (1–3), integrin α4β1 and calponin (4–6), and integrin α9β1 and calponin (7–9) in mouse aorta sections. Scale bar indicates 50 μm.
Figure S3: SVEP1, integrin α4β1 and α9β1 are expressed on endothelial cells
(A) HUVECs were fixed and stained with 3 μg/mL rabbit lgG, or (B) 2 μG/ml mouse IgG and appropriate secondary antibody, Dual fluorescent staining of SVEP1 (C & E) and integrin α4β1 (D & E), AND SVEP1 (F & H) and integrin α9β1 (G & H). Scale bar indicates 50 μm.
Figure S4: Antibody controls for ICC staining
(A) Aortic section stained for IHC had no primary antibodies added. iVSMCs were stained with 3 μg/mL rabbit lgG (B), or 2 μg/mL mouse lgG (C) and appropriate secondary antibody. Aortic sections were imaged on a confocal microscope using the same laser settings as used for staining. Sections had no antibodies added (D), no primary antibodies (E), Mouse lgG at 2 μg/mL (F) or rabbit lgG at 3 μg/mL (G). Scale bar indicates 100 μm.
Figure S5: iPSC differentiation into iVSMC protocol
**A)** Timeline of iPSC differentiation into iVSMCs **B)** qPCR data showing (1) pluripotency, (2) primitive streak and (3) mesodermal gene expression (n = 4). Transmitted light (4) and immunofluorescence images showing OCT4 (pluripotency) (5) and brachyury (primitive streak) (6) protein expression from day 0 to day 4 of iPSC differentiation, scale bar indicates 400 μm. (7) Flow cytometric analysis of CD140b+ cells at day 8 (n = 4, mean ± SD). **C)** iVSMC progenitor cells were cultured in media supplemented with PDGF‐BB (10 ng/mL) and TGFβ (2 ng/mL) for 12 days before characterisation. qPCR expression of (1) ACTA2, (2) CNN1, and (3) SMTHLN (n = 4), and protein expression data of (4) smooth muscle α actin, (5) calponin and (6) smoothelin, scale bar indicates 100 μm. **D)** iVSMC contractility was measured by (1) collagen gel contraction and (2) intracellular Ca^2+^ elevation to several vasoconstrictors, with the relative contractility of cultured human aortic smooth muscle cells (AoSMC) to the same vasoconstrictors is shown in (3). Data are respresented as means ±SEM.
Figure S6: Quantification of ITGA4. ITGA9 and SVEP1 siRNA treatment
iVSMCs were transfected with 100 nM of non‐targeting control (NTC), ITGA4, ITGA9, or SVEP1 siRNA for 48 hrs. Cells were lysed and relative gene expression of *ITGA4* (A, n = 4), *ITGA9* (B, n = 3), and *SVEP1* (C, n = 3) were measured by qRT‐PCR and normalised to gene expression in NTC‐transfected cells. iVSMCs were fixed and integrin α4 (D), α9 (E), and SVEP1 (F) protein expression was visualised by immunocytochemistry (scale bar indicates 50 μm). iVSMCs were lysed and integrin α4 (G, n = 3) and α9 (H, n = 3) protein expression was quantified by densitometry and normalised to protein levels in NTC‐transfected cells. Data are represented as means ± SD.
Figure S7. Generation of SVEP1 knockoutiPSCs.
(A) Truncated schematic of SVEP1 gene structure. CRISPR guide RNA targeted region in exon 1 of SVEP1. (B) SVEP1 sequencing from parental iPSC line. (C) SVEP1 exon 1 sequencing from SVEP1^−/−^ iPSC line with deletion event shown by the black triangle.
**Figure S8:SVEP** ^−/−^ **iVSMCs display elevated [Ca** ^**2+**^ **]**_**i**_ **to ET‐1 or carbachol.**
iVSMCs differentiated from parental wild type (WT) isotype control iPSCs or SVEP ^−/−^ knockout (KO) iPSCs were loaded with Fluo3 prior to vasoconstrictior challenge for 45 secs. Maximal fluorescence signal (F/F _0_) are shown for (A) ET‐1 (50 nM, n = 8), and (B) Cch (100 μM, WT n = 10, KO n = 9). Data are represented as means ± SD, * P < .05, unpaired *t*‐test.
**Figure S9: Simultaneous inhibition of SVEP1 and integrin α4 or α9 does not enhance [Ca** ^**2+**^ **]**_**i**_ **elevation to a panel of vasoconstrictors**
(A) iVSMCs were treated with non‐targeting control (NTC), ITGA4, and SVEP1 siRNA, (B) NTC, ITGA9 and SVEP1 siRNA for 48 hrs prior to Fluo3 loading and ET‐1 (50 nM) challenge for 45 secs. (C) iVSMCs were treated with NTC, ITGA4, ITGA9 and SVEP1 siRNA, for 48 hrs prior to Fluo3 loading and carbachol (100 μM) challenge for 45 secs. Maximal fluorescence signal (*F/F*_*O*_) are shown (A NTC n = 7. SVEP1 n = 6, ITGA4, SVEP1&ITGA4 n = 5, B NTC n = 8, SVEP1 n = 5, ITGA9 n = 7, SVEP1&ITGA9 n = 6, C all conditions n = 5). (D) iVSMCs differentiated from parental wild type (WT) isotype control iPSCs or SVEP1 knockout (KO) iPSCs were treated with the dual integrin α4β1 – α9β1 inhibitor BOP for 1 hr prior to Fluo3 loading and ET‐1 (50 nM) challenge for 45 secs. Maximal fluorescence signal (*F/F*_*0*_) are shown (WT n = 13, BOP, KO n = 14, KNO&BOP n = 10). Data are represented as means ±SD, *P < .05, one‐way ANOVA followed by Turkeys post hoc test.
Figure S10: Aortic contraction to vasoconstrictors, direct activation or calcium channel blockage.
(A) Aortas from Svep1 ^+/−^ mice or littermate controls were stimulated with PE and force generation was recorded by wire myography (+/+ n = **1**1, +/− n = 15).
(B) Aortas from C5BL/6 J mice were pre‐incubated with integrin α4 and α9 blocking antibodies overnight and incubated with the VGCC blocker nifedipine (NF) for minutes prior to U46619 application (n = 4). (C) Direct activation of voltage gated calcium channels (VGCCs) was induced by application of a high K^+^ solution in aortas from Sveop1 ^+/−^ mice or littermate controls (+/+ n = 9, +/− n = 11) and (D) aortas from C54BL/6 J, mice pre‐incubated with the dual integrin α4/α9 inhibitor BOP overnight (ns n = 6, BOP n = 9), with the force generation recorded by wire myography. Data are represendted as means ± SD, * *P* < .05, mixed‐effects models.

## Data Availability

The data supporting the findings in this study are available from the corresponding author upon reasonable request.
