# Supplementary material for: The integrin ligand SVEP1 regulates GPCR‐mediated vasoconstriction via integrins α9β1 and α4β1
Source: Br J Pharmacol. 2022 Aug 2;179(21):4958–73. doi: 10.1111/bph.15921 (PMC9805129; doi:10.1111/bph.15921)
Supplement: Supplementary file 1 — Table S1: Primer sequences Table S2: Antibody suppliers and catalogue numbers [file BPH-179-4958-s001.docx]

| **Gene** | **Forward Primer** | **Reverse Primer** |
| --- | --- | --- |
| ***RPLP0*** | TCGACAATGGCAGCATCTAC | GCCTTGACCTTTTCAGCAAG |
| ***ACTA2*** | CTGTTCCAGCCATCCTTCAT | CCGTGATCTCCTTCTGCATT |
| ***CNN1*** | ACATTTTTGAGGCCAACGAC | ACTTCACTCCCACGTTCACC |
| ***SMTHN*** | CGAGTGAACAAAGCACCAGA | ATGAGCTTCCGCTCTTCAAA |
| ***.OCT4*** | CGAGAGGATTTTGAGGCTGC | CGAGGAGTACAGTGCAGTGA |
| ***NANOG*** | CCTCCTCCCATCCCTCAT | GGATGGGCATCATGGAAA |
| ***SVEP1*** | ATTCCTGCAACAGAGGCTAC | CAGGGCATGGATTTGGTTTG |
| ***TAGLN*** | QuantiTect Primers | Cat: QT00072247 |
| ***ITGA4*** | QuantiTect Primers | Cat: QT00060627 |
| ***ITGA9*** | QuantiTect Primers | Cat:QT00018921 |

Table S1: Primer sequences

| **Target** | **Company** | **Cat. Number** | **Protocol** | **Species** | **Isotype** | **Dilution** |
| --- | --- | --- | --- | --- | --- | --- |
| IgG isotype control | R&D Systems | AB-105-C | ICC | Rabbit | IgG |  |
| IgG isotype control | Thermo-fisher | #31903 | ICC | Mouse | IgG |  |
| IgG isotype control | Thermo-fisher | #31245 | ICC | Goat | IgG |  |
| Anti-SVEP1 | AbCam | ab121677 | ICC | Rabbit | IgG | 75 |
| Anti mouse-integrin alpha 9 | R&D Systems | AF3827 | ICC | Goat | IgG | 200 |
| Anti-integrin alpha 9 | AbCam | ab27947 | ICC | Mouse | IgG1 | 100 |
| Anti-integrin alpha 4 | Santa Cruz | SC365209 | ICC, WB | Mouse | IgG2 | 200/1000 |
| Anti-OCT4 | Abcam | ab19857 | ICC | Rabbit | IgG | 200 |
| Anti-brachyury | Abcam | ab209665 | ICC | Rabbit | Animal Free | 200 |
| Anti-actin α-smooth muscle | Sigma | A2547 | ICC | Mouse | IgG2 | 250 |
| Anti-SM22α | AbCam | ab14106 | ICC | Rabbit | IgG | 250 |
| Anti-calponin | AbCam | ab46794 | ICC | Rabbit | Animal free | 250 |
| Anti-smoothelin | Santa Cruz | SC376902 | ICC | Mouse | IgG2 | 200 |
| Alexa Fluor 488 goat anti-rabbit | Thermo-fisher | A11034 | ICC | Goat | IgG | 500 |
| Alexa Fluor 555 goat anti-mouse | Thermo-fisher | A21050 | ICC | Goat | IgG | 500 |
| Alexa Fluor 595 donkey anti-goat | AbCam | ab150132 | ICC | Donkey | IgG | 500 |
|  |  |  |  |  |  |  |
| Anti-GFP | Thermo-fisher | A11120 | IP | Mouse | IgG2 | 1000 |
|  |  |  |  |  |  |  |
| CD140b-APC | Miltenyi Biotech | 130-105-322 | FC | Human | IgG | 50 |
|  |  |  |  |  |  |  |
| Anti-FLAG | Sigma | F3165 | WB | Mouse | IgG | 1000 |
| Anti-integrin alpha 9 | AbCam | ab140599 | WB | Rabbit | Animal free | 500 |
| Anti-beta-actin mouse monoclonal | Santa Cruz | SC47778 | WB | Mouse | IgG | 5000 |
| Anti-Rabbit IgG HRP-linked | Cell signalling technology | 7074S | WB | Goat | IgG | 5000 |
| Anti-mouse IgG HRP-linked | Cell signalling technology | 7076S | WB | Horse | IgG | 5000 |
|  |  |  |  |  |  |  |
| Rat IgG2b antibody | Biorad | MCA6006GA | Myography |  |  |  |
| Hamster IgG clone | Biorad | OBT1107 | Myography |  |  |  |
| Anti-CD49b (MCA1230) | Biorad | MCA1230GA | Myography | Rat |  |  |
| Anti-mouse integrin α9 (55A2C) | Kind gift from Prof. Shigeyuki Kon | | Myography | Hamster |  |  |

Table S2: Antibody suppliers and catalogue numbers
