# Supplementary material for: The integrin ligand SVEP1 regulates GPCR‐mediated vasoconstriction via integrins α9β1 and α4β1
Source: Br J Pharmacol. 2022 Aug 2;179(21):4958–73. doi: 10.1111/bph.15921 (PMC9805129; doi:10.1111/bph.15921)
Supplement: Supplementary file 2 — Figure S1: SVEP1 and integrin expression in the vasculature (A) SVEP1, (B) ITGA4 and (C) ITGA9 mRNA expression was measured using qRT‐PCR in human coronary artery endothelial cells (CAEC) and vascular smooth muscle cells (VSMC). Results are normalised to the reference gene RPLPO. Data are represented as means± SD,n = 3. Figure S2.SVEP1 integrin α4β1 and integrin α9β1 are localised to smooth muscle cell layer Dual fluorescent staining of SVEP1 and smooth muscle α‐actin (1–3), integrin α4β1 and calponin (4–6), and integrin α9β1 and calponin (7–9) in mouse aorta sections. Scale bar indicates 50 μm. Figure S3: SVEP1, integrin α4β1 and α9β1 are expressed on endothelial cells (A) HUVECs were fixed and stained with 3 μg/mL rabbit lgG, or (B) 2 μG/ml mouse IgG and appropriate secondary antibody, Dual fluorescent staining of SVEP1 (C & E) and integrin α4β1 (D & E), AND SVEP1 (F & H) and integrin α9β1 (G & H). Scale bar indicates 50 μm. Figure S4: Antibody controls for ICC staining (A) Aortic section stained for IHC had no primary antibodies added. iVSMCs were stained with 3 μg/mL rabbit lgG (B), or 2 μg/mL mouse lgG (C) and appropriate secondary antibody. Aortic sections were imaged on a confocal microscope using the same laser settings as used for staining. Sections had no antibodies added (D), no primary antibodies (E), Mouse lgG at 2 μg/mL (F) or rabbit lgG at 3 μg/mL (G). Scale bar indicates 100 μm. Figure S5: iPSC differentiation into iVSMC protocol A) Timeline of iPSC differentiation into iVSMCs B) qPCR data showing (1) pluripotency, (2) primitive streak and (3) mesodermal gene expression (n = 4). Transmitted light (4) and immunofluorescence images showing OCT4 (pluripotency) (5) and brachyury (primitive streak) (6) protein expression from day 0 to day 4 of iPSC differentiation, scale bar indicates 400 μm. (7) Flow cytometric analysis of CD140b+ cells at day 8 (n = 4, mean ± SD). C) iVSMC progenitor cells were cultured in media supplemented with PDGF‐BB (10 ng/mL) and [file BPH-179-4958-s002.pdf]

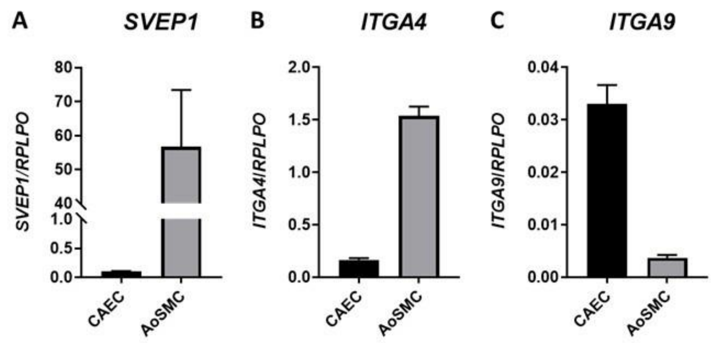

Supplemental Figure 1: SVEP1 and integrin expression in the vasculature
(A) SVEP1, (B) ITGA4 and (C) *ITGA9* mRNA expression was measured using qRT-PCR in human coronary artery endothelial cells (CAEC) and vascular smooth muscle cells (VSMC). Results are normalised to the reference gene *RPLP0*. Data are represented as means ± SD, n=3.

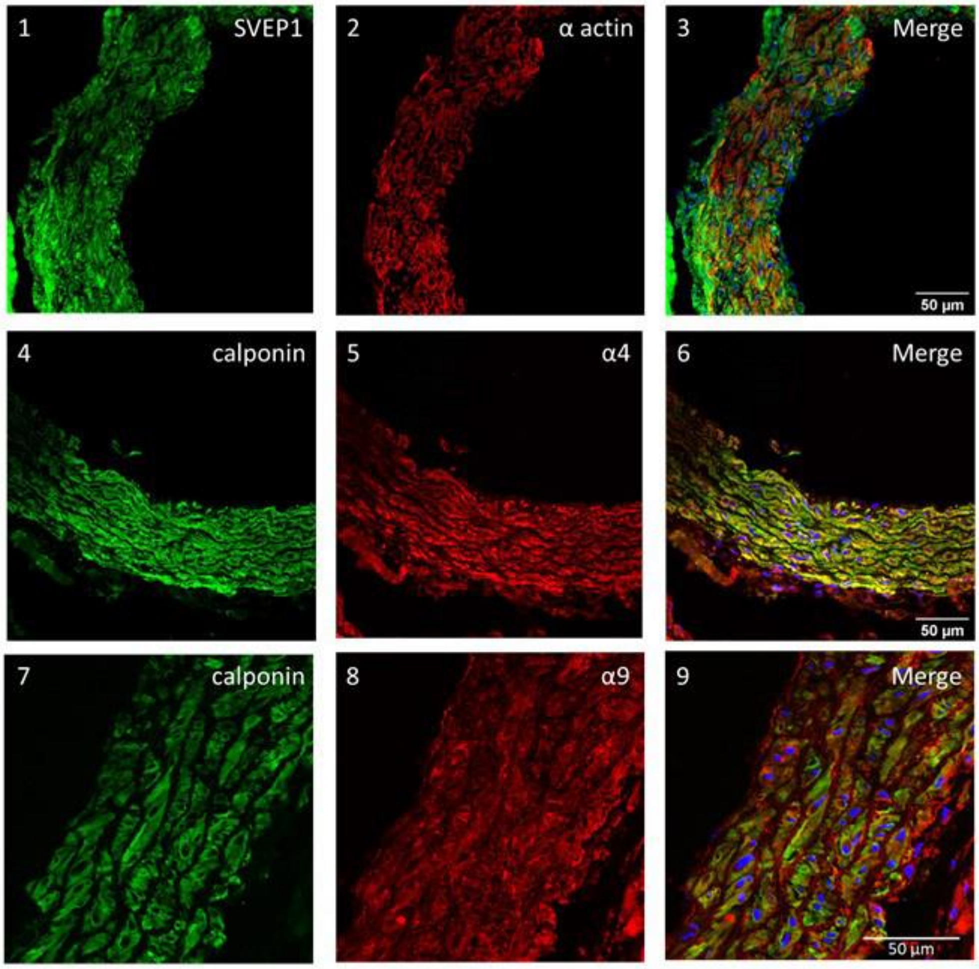

Supplemental Figure 2. SVEP1, integrin α4β1 and integrin α9β1 are localised to smooth muscle cell layer

Dual fluorescent staining of SVEP1 and smooth muscle α-actin (1-3), integrin α4β1 and calponin (4-6), and integrin α9β1 and calponin (7-9) in mouse aorta sections. Scale bar indicates 50µm.

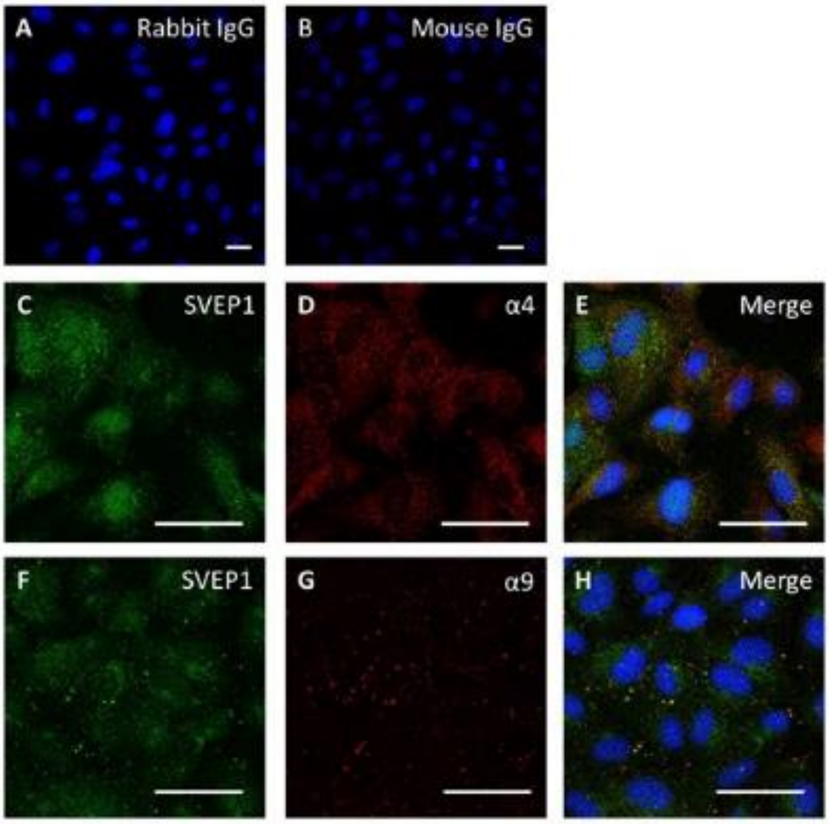

Supplemental Figure 3: SVEP1, integrin α4β1 and α9β1 are expressed on endothelial cells

(A) HUVECs were fixed and stained with 3 μg/ml rabbit IgG, or (B) 2 μg/ml mouse IgG and appropriate secondary antibody. Dual fluorescent staining of SVEP1 (C & E) and integrin α4β1 (D & E), and SVEP1 (F & H) and integrin α9β1 (G & H). Scale bar indicates 50 μm.

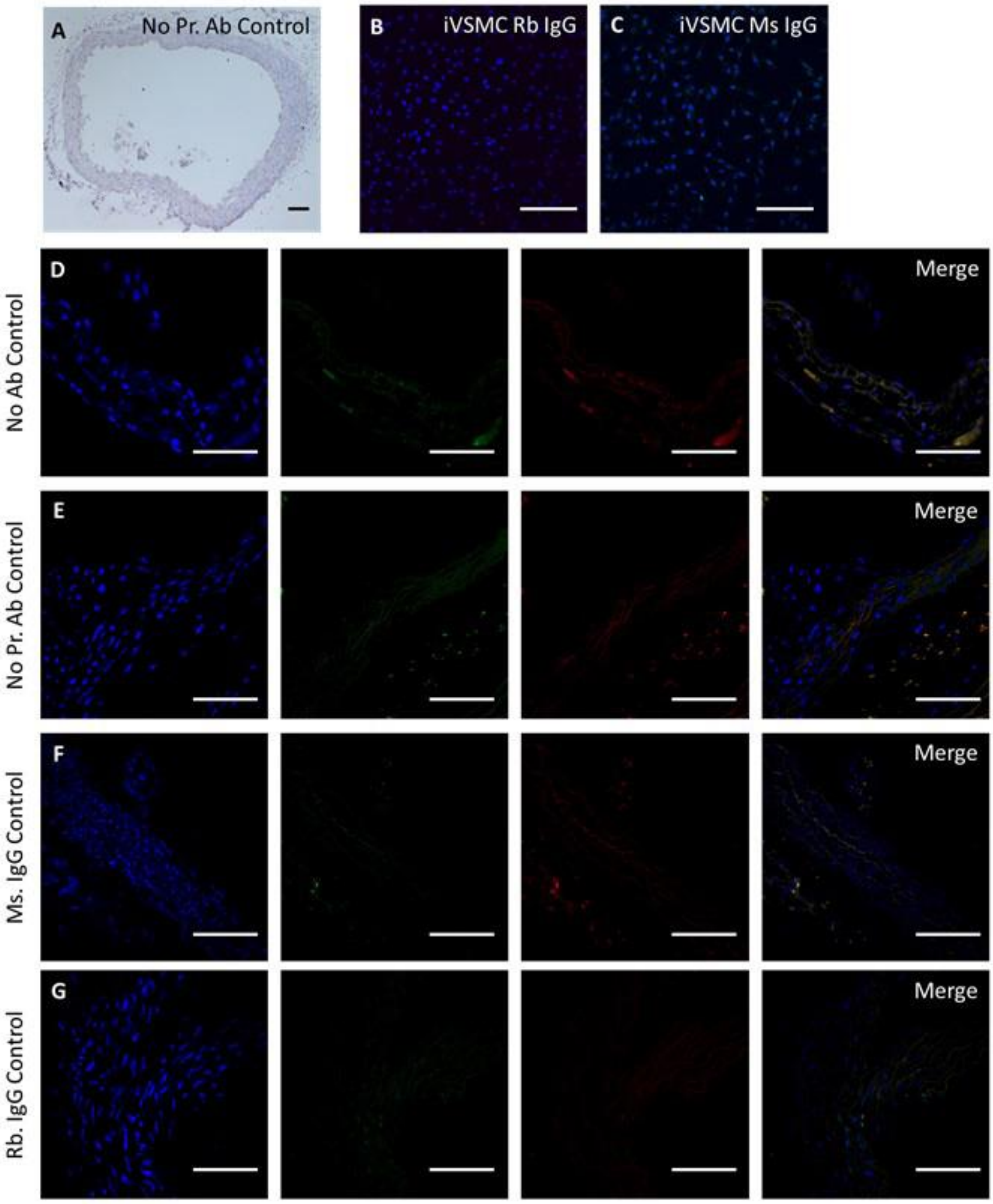

Supplemental Figure 4: Antibody controls for ICC staining

(A) Aortic section stained for IHC had no primary antibodies added. iVSMCs were stained with 3 µg/ml rabbit IgG (B), or 2 µg/ml mouse IgG (C) and appropriate secondary antibody. Aortic sections were imaged on a confocal microscope using the same laser settings as used for staining. Sections had no antibodies added (D), no primary antibodies (E), Mouse IgG at 2 µg/ml (F) or rabbit IgG at 3 µg/ml (G). Scale bar indicates 100 µm.

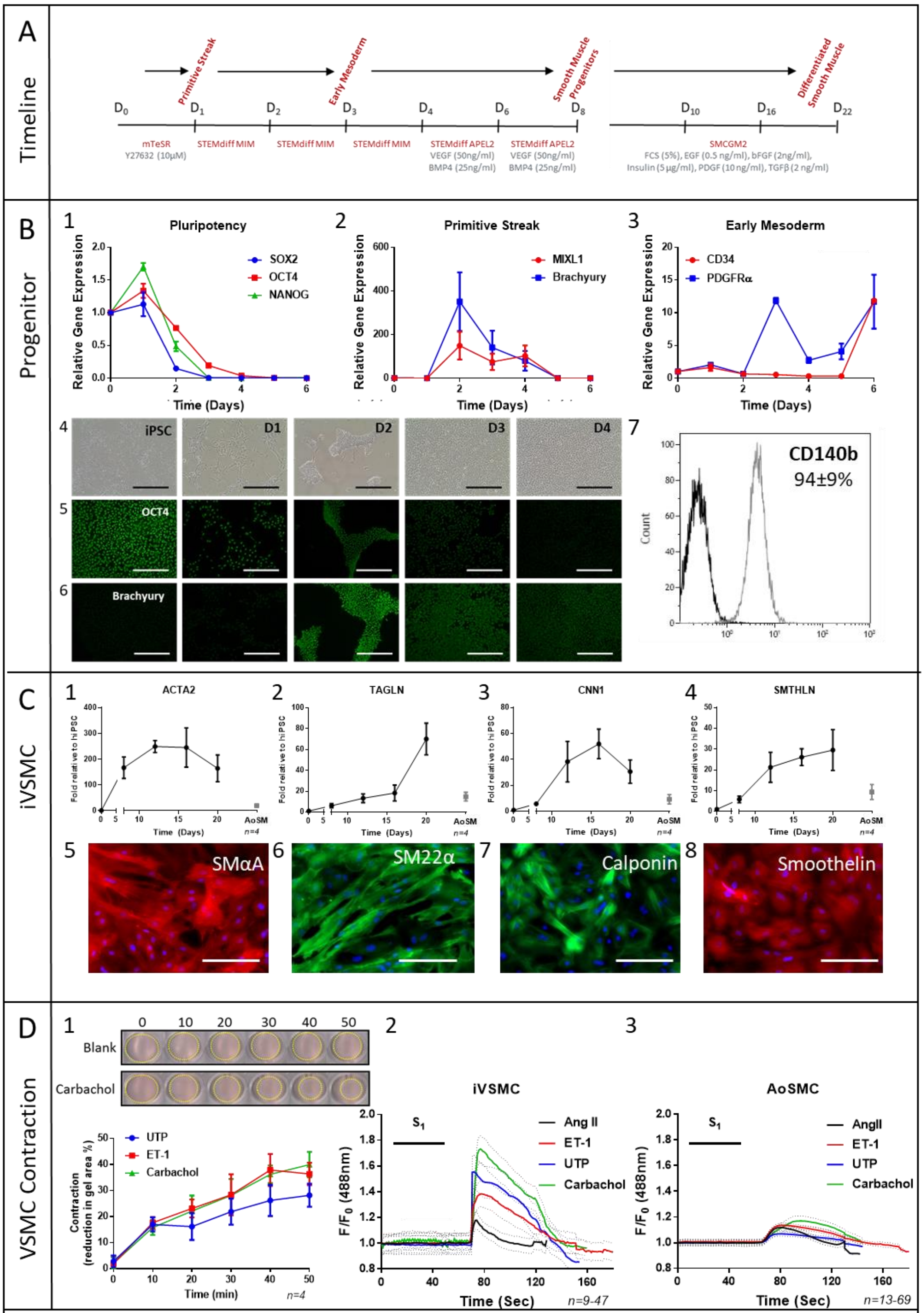

Supplemental Figure 5: iPSC differentiation into iVSMC protocol

A) Timeline of iPSC differentiation into iVSMCs **B**) qPCR data showing (1) pluripotency, (2) primitive streak and (3) mesodermal gene expression (n=4). Transmitted light (4) and immunofluorescence images showing OCT4 (pluripotency) (5) and brachyury (primitive streak) (6) protein expression from day 0 to day 4 of iPSC differentiation, scale bar indicates 400 µm. (7) Flow cytometric analysis of $CD140b^+$ cells at day 8 (n=4, mean±SD). **C**) iVSMC progenitor cells were cultured in media supplemented with PDGF-BB (10 ng/ml) and TGFβ (2 ng/ml) for 12 days before characterisation. qPCR expression of (1) ACTA2, (2) CNN1, and (3) SMTHLN (n=4), and protein expression data of (4) smooth muscle α actin, (5) calponin and (6) smoothelin, scale bar indicates 100 µm. **D**) iVSMC contractility was measured by (1) collagen gel contraction and (2) intracellular Ca^{2+} elevation to several vasoconstrictors, with the relative contractility of cultured human aortic smooth muscle cells (AoSMC) to the same vasoconstrictors is shown in (3). Data are represented as means ± SEM.

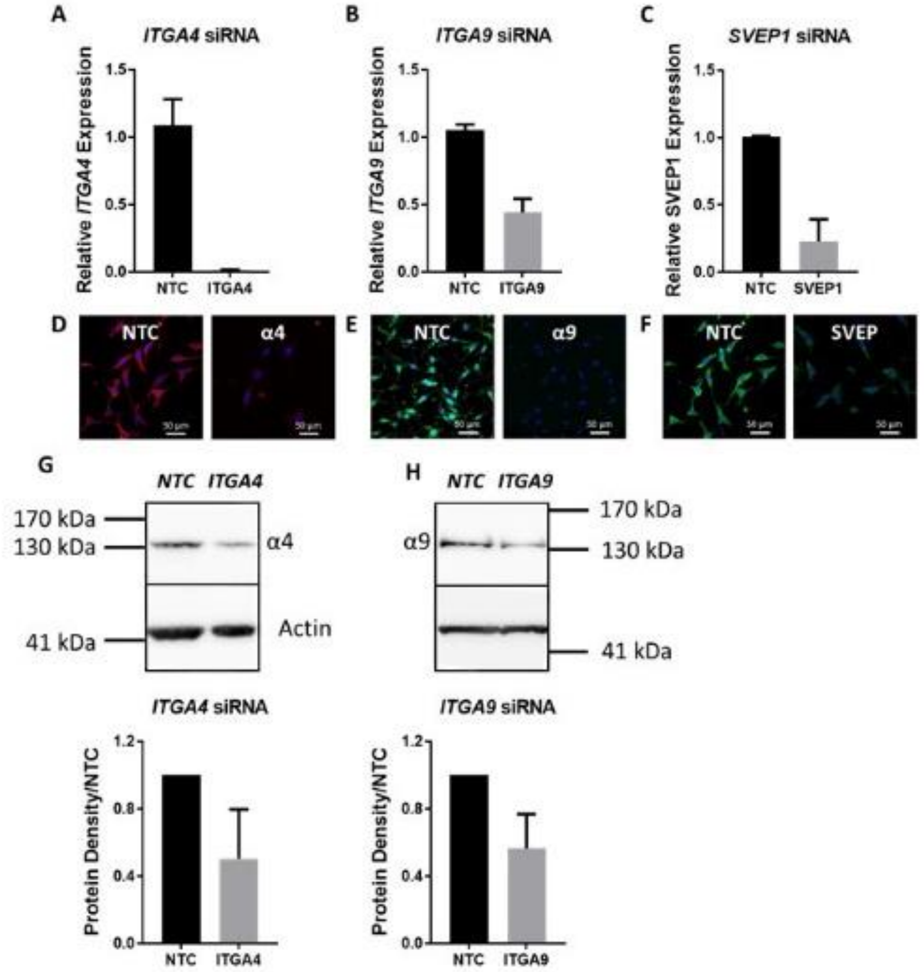

Supplemental Figure 6: Quantification of ITGA4, ITGA9 and SVEP1 siRNA treatment

iVSMCs were transfected with 100 nM of non-targeting control (NTC), ITGA4, ITGA9, or SVEP1 siRNA for 48 hrs. Cells were lysed and relative gene expression of *ITGA4* (A, n=4), *ITGA9* (B, n=3), and *SVEP1* (C, n=3) were measured by qRT-PCR and normalised to gene expression in NTC-transfected cells. iVSMCs were fixed and integrin α4 (D), α9 (E), and SVEP1 (F) protein expression was visualised by immunocytochemistry (scale bar indicates 50 μm). iVSMCs were lysed and integrin α4 (G, n=3), and α9 (H, n=3) protein expression was quantified by densitometry and normalised to protein levels in NTC-transfected cells. Data are represented as means ± SD.

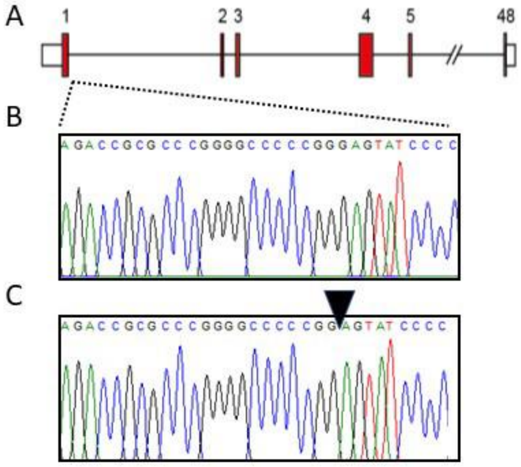

Supplemental Figure 7. Generation of *SVEP1* knockout iPSCs.
(A) Truncated schematic of *SVEP1* gene structure. CRISPR guide RNA targeted region in exon 1 of *SVEP1*. *(B) SVEP1* sequencing from parental iPSC line. (C) *SVEP1* exon 1 sequencing from SVEP1$^{-/-}$ iPSC line with deletion event shown by the black triangle.

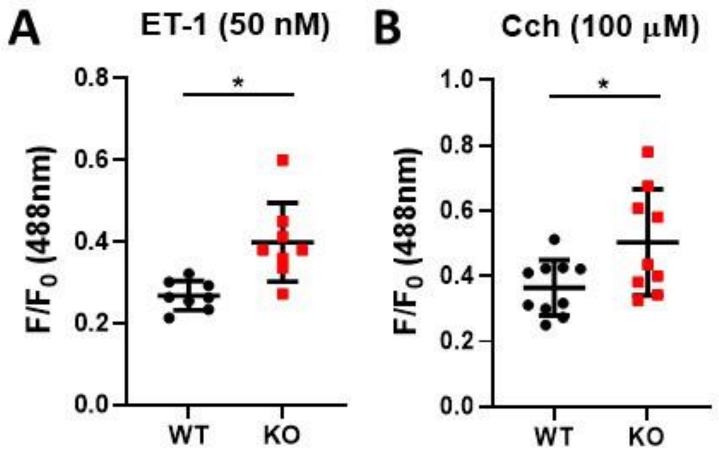

Supplemental Figure 8: SVEP1$^{-/-}$ iVSMCs display elevated $[Ca^{2+}]_i$ to ET-1 or carbachol.

iVSMCs differentiated from parental wild type (WT) isotype control iPSCs or *SVEP1$^{-/-}$* knockout (KO) iPSCs were loaded with Fluo3 prior to vasoconstrictior challenge for 45 secs. Maximal fluorescence signal (F/F_0) are shown for (A) ET-1 (50 nM, n=8), and (B) Cch (100 μM, WT n=10, KO n=9). Data are represented as means ± SD, * $P < 0.05$, unpaired *t*-test.

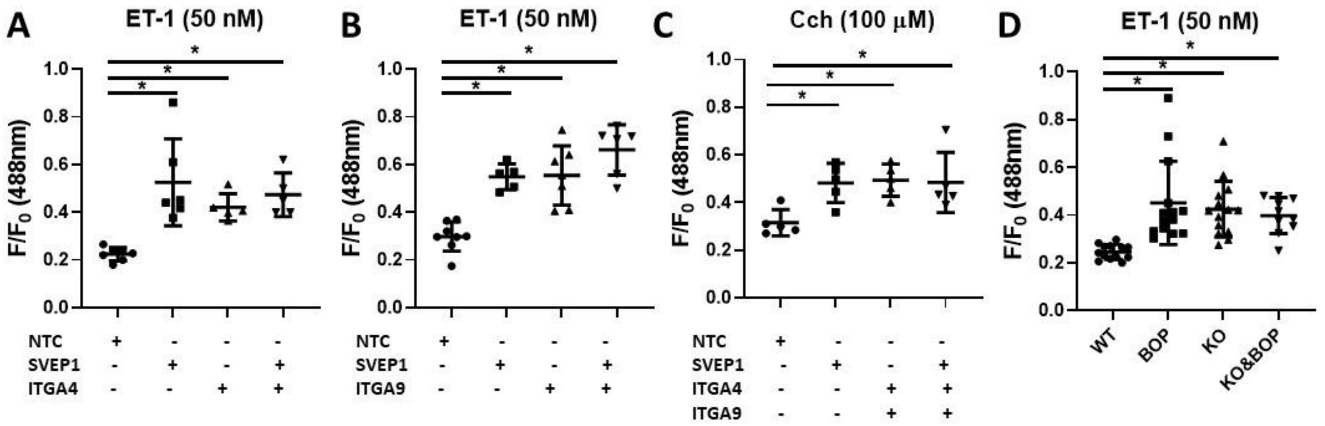

Supplemental Figure 9: Simultaneous inhibition of SVEP1 and integrin α4 or α9 does not enhance $[Ca^{2+}]_i$ elevation to a panel of vasoconstrictors
(A) iVSMCs were treated with non-targeting control (NTC), ITGA4, and SVEP1 siRNA, (B) NTC, ITGA9 and SVEP1 siRNA for 48 hrs prior to Fluo3 loading and ET-1 (50 nM) challenge for 45 secs. (C) iVSMCs were treated with NTC, ITGA4, ITGA9 and SVEP1 siRNA, for 48 hrs prior to Fluo3 loading and carbachol (100 μM) challenge for 45 secs. Maximal fluorescence signal (F/F_0) are shown (A NTC n=7, SVEP1 n=6, ITGA4, SVEP1&ITGA4 n= 5, B NTC n=8, SVEP1 n=5, ITGA9 n=7, SVEP1&ITGA9 n=6, C all conditions n=5). (D) iVSMCs differentiated from parental wild type (WT) isotype control iPSCs or SVEP1 knockout (KO) iPSCs were treated with the dual integrin α4β1-α9β1 inhibitor BOP for 1 hr prior to Fluo3 loading and ET-1 (50 nM) challenge for 45 secs. Maximal fluorescence signal (F/F_0) are shown (WT n=13, BOP, KO n=14, KO&BOP n=10). Data are represented as means ± SD, * $P < 0.05$, one-way ANOVA followed by Tukey's post hoc test.

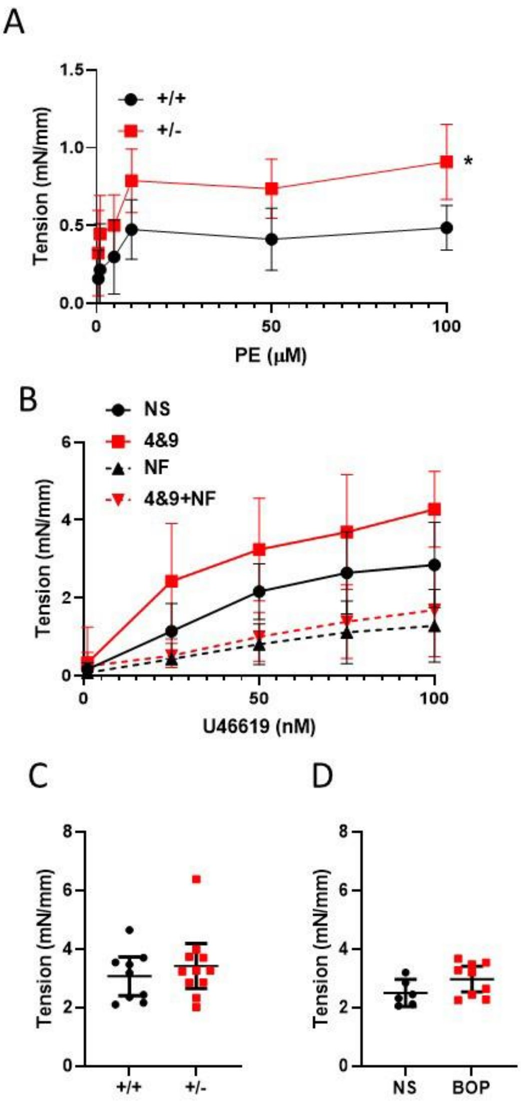

Supplemental Figure 10: Aortic contraction to vasoconstrictors, direct activation or calcium channel blockage.

(A) Aortas from *Svep1*$^{+/-}$ mice or littermate controls were stimulated with PE and force generation was recorded by wire myography (+/+ n=11, +/- n=15). (B) Aortas from C57BL/6J mice were pre-incubated with integrin α4 and α9 blocking antibodies overnight and incubated with the VGCC blocker nifedipine (NF) for 30 minutes prior to U46619 application (n=4). (C) Direct activation of voltage gated calcium channels (VGCCs) was induced by application of a high K^+ solution in aortas from *Svep1*$^{+/-}$ mice or littermate controls (+/+ n=9, +/- n=11) and (D) aortas from C57BL/6J mice pre-incubated with the dual integrin α4/α9 inhibitor BOP overnight (ns n=6, BOP n=9), with force generation recorded by wire myography. Data are represented as means ± SD, * $P < 0.05$, mixed-effects models.
